# The association between neutrophil percentage to albumin ratio and progression-free survival and overall survival in colorectal cancer patients: a retrospective cohort study

**DOI:** 10.3389/fnut.2025.1589854

**Published:** 2025-07-10

**Authors:** Hailun Xie, Lishuang Wei, Shuangyi Tang, Jialiang Gan

**Affiliations:** ^1^Department of Gastrointestinal and Gland Surgery, The First Affiliated Hospital, Guangxi Medical University, Nanning, China; ^2^Guangxi Key Laboratory of Enhanced Recovery After Surgery for Gastrointestinal Cancer, Nanning, China; ^3^Department of Geriatric Respiratory Disease Ward, The First Affiliated Hospital, Guangxi Medical University, Nanning, China; ^4^Department of Pharmacy, The First Affiliated Hospital, Guangxi Medical University, Nanning, China; ^5^Department of Colorectal and Anal Surgery, The First Affiliated Hospital, Guangxi Medical University, Nanning, China

**Keywords:** neutrophil percentage, albumin, colorectal cancer, progression-free survival, overall survival

## Abstract

**Background:**

The neutrophil percentage-to-albumin ratio (NPAR) is a promising indicator for predicting outcomes in various cancers. However, its prognostic value in colorectal cancer (CRC) is still underexplored. This study aimed to investigate the relationship between NPAR and progression-free survival (PFS) as well as overall survival (OS) in CRC patients.

**Methods:**

We conducted a retrospective cohort study involving 1,339 CRC patients who underwent surgical resection. The Kaplan-Meier method was utilized to plot survival curves for PFS and OS. Cox proportional hazards regression analysis assessed the relationship between NPAR and survival outcomes. The nomograms that included NPAR and other significant prognostic factors were developed to predict 1-, 3-, and 5-year survival rates.

**Results:**

Patients with high NPAR (≥1.62) experienced significantly worse PFS and OS compared to those with low NPAR (<1.62) (PFS: 47.4% vs. 63.1%, *p* < 0.001; OS: 50.1% vs. 65.9%, *p* < 0.001). Compared to other relevant markers, NPAR exhibited strong prognostic predictive efficacy. Multivariate Cox regression analysis identified high NPAR as an independent predictor of poor PFS (hazard ratio [HR] = 1.671, 95% Confidence Interval [CI]: 1.142–2.444, *p* = 0.008) and OS (HR = 2.697, 95% CI: 1.761–4.130, *p* < 0.001). The NPAR-based nomograms demonstrated high predictive accuracy and received favorable evaluations in the internal validation cohort.

**Conclusion:**

Preoperative NPAR is a promising indicator for predicting PFS and OS in CRC patients. The NPAR-based nomogram offers a practical tool for personalized survival prediction and may assist in clinical decision-making.

## Introduction

Colorectal cancer (CRC) is one of the common malignancies that seriously threaten human health worldwide, with a high incidence and mortality rate. In recent years, the incidence and mortality of CRC have been on the rise due to global population aging and changes in lifestyle and dietary habits (1). According to the latest global cancer statistics, CRC ranks third in terms of new cases and second in terms of mortality among all cancers, severely impacting the quality of life and life expectancy of patients (2). In China, the incidence of CRC is also alarming, with a trend toward younger age groups, imposing a heavy burden on patients' families and society (3–5). Although certain progress has been made in the diagnosis and treatment of CRC, such as the continuous improvement of surgical techniques, the renewal of chemotherapy drugs, and the emergence of targeted therapy and immunotherapy, many patients are still diagnosed at the middle or advanced stages, missing the optimal treatment opportunity and resulting in a poor prognosis (6, 7). The 5-year survival rate of CRC patients in China is only 56.9%, which is relatively low among the surrounding Asia Pacific regions (8, 9). Therefore, the search for reliable tumor prognostic indicators is of great significance for predicting the disease progression of CRC patients, evaluating treatment efficacy, and formulating personalized treatment plans.

Traditional prognostic assessment indicators, such as the tumor node metastasis (TNM) staging of tumors, pathological type, and degree of differentiation, although widely used in clinical practice, have certain limitations in predicting the individual prognosis of patients. They cannot comprehensively reflect the biological behavior of tumors and the overall condition of patients (10–12). Thus, the search for new and more accurate prognostic markers has become a research hotspot. In recent years, an increasing number of studies have shown that systemic inflammation and nutritional status have been increasingly recognized as critical factors influencing cancer progression and patient outcomes (13–17). Recently, a serological inflammation-nutrition-related index, the neutrophil percentage to albumin ratio (NPAR), has been developed. It has been reported as an effective prognostic marker for predicting the risk of death related to cardiovascular diseases, stroke, bladder cancer, and oral cancer (18–20). Neutrophils, as key mediators of inflammation, play a crucial role in cancer progression. They promote tumor growth and metastasis through various mechanisms, including the release of reactive oxygen species (ROS) and suppression of anti-tumor immunity (21, 22). However, emerging evidence highlights their context-dependent anti-tumor functions. For instance, specific neutrophil subsets (e.g., N1-polarized neutrophils) can directly kill tumor cells via cytotoxic molecules like elastase and myeloperoxidase or indirectly enhance anti-tumor immunity by facilitating antigen presentation and activating cytotoxic T lymphocytes (23). Neutrophil extracellular traps (NETs), while implicated in metastatic dissemination, may also restrict early tumor spread by trapping circulating tumor cells (24). Thus, neutrophils exhibit a dual role in cancer, governed by their phenotypic plasticity and microenvironmental cues. Conversely, serum albumin, a marker of nutritional status, is associated with better outcomes in cancer patients, as low albumin levels often reflect malnutrition and systemic inflammation (25, 26). The NPAR, by integrating these two factors, provides a comprehensive assessment of the inflammatory and nutritional status of cancer patients.

Previous studies have shown that NPAR has prognostic value in various cancers, such as oral cavity cancer, breast cancer, and bladder cancer (27–29). However, currently, research on the prognostic assessment of NPAR in CRC is still lacking, and its specific mechanism of action and clinical application value remain incompletely clear. In CRC, systemic inflammation and malnutrition are closely linked to disease progression and immune evasion. Neutrophils in CRC exhibit unique interactions with the tumor microenvironment, while hypoalbuminemia reflects CRC-associated metabolic dysregulation and cachexia. These CRC-specific pathways amplify NPAR's dual role in capturing inflammatory burden and nutritional status, making it particularly relevant for CRC prognosis. Therefore, this study aims to conduct a retrospective analysis of the clinical data of CRC patients to explore the relationship between NPAR and the progression-free survival (PFS) and overall survival (OS) of CRC patients, providing new ideas and evidence for the prognostic assessment and clinical treatment of CRC.

## Materials and methods

### Population

We conducted a retrospective analysis of 1,339 CRC patients who underwent surgical resection at the First Affiliated Hospital of Guangxi Medical University between 2015 and 2017. The inclusion criteria were as follows: histologically confirmed CRC; availability of complete data regarding albumin, neutrophil percentage, and other relevant clinicopathological factors; and age ≥ 18 years. Patients were excluded if they had received neoadjuvant chemotherapy before surgery, had other concurrent malignancies, suffered from pre-existing autoimmune diseases, or had acute or chronic inflammatory diseases that could affect the levels of neutrophils or albumin at the time of data collection. This study was approved by the Institutional Review Board of the First Affiliated Hospital of Guangxi Medical University, and informed consent was obtained from all participants.

### Data collection

In this study, we comprehensively collected the clinicopathological data of patients through the hospital's electronic medical record system. Specifically, it included patients' basic information such as name, gender, age, height, weight, and contact information. This information was helpful for analyzing and understanding the general conditions of patients. Regarding tumor-related information, key details were recorded in detail, including the tumor location (colon cancer or rectal cancer), TNM staging [accurately determined according to the 8th Edition of the American Joint Committee on Cancer (AJCC) tumor staging system], tumor size, histological type (such as adenocarcinoma, mucinous adenocarcinoma, undifferentiated carcinoma, etc.), degree of differentiation (well/moderately differentiated, poorly differentiated), and the presence of perineural invasion and vascular invasion. Laboratory-related factors included neutrophil percentage, albumin levels, and other routine blood test results. The blood samples were obtained from patients within 7 days before surgery for testing. NPAR was calculated using the formula: NPAR = Neutrophil percentage (%)/Albumin (g/L). Neutrophil (10^9^/L) to lymphocyte ratio (NLR) (10^9^/L) is defined as neutrophil divided by lymphocyte, Platelet (10^9^/L) to lymphocyte (10^9^/L) ratio (PLR) is defined as platelet divided by lymphocyte, and prognostic nutritional index (PNI) is defined as serum albumin (g/L) + 5 × lymphocytes (10^9^/L). Previous published literatures have confirmed that these prognostic indicators serve as effective prognostic markers for predicting the outcomes of CRC patients (30–34).

### Follow-up

In this study, we conducted long term follow up of patients through a combination of telephone follow up and outpatient reexamination. Patients were followed up every 3 months for the first 2 years, then every 6 months for the next 3 years, and annually thereafter. The follow up content included a detailed inquiry about patients' symptoms and signs to check for any signs of recurrence or metastasis. Meanwhile, patients were required to undergo necessary examinations, such as blood routine tests, biochemical examinations, tumor marker tests, colonoscopy, and imaging examinations like computed tomography (CT) or magnetic resonance imaging (MRI), to comprehensively assess their condition. The primary endpoints were PFS and OS. PFS was defined as the time from surgery to the first occurrence of local recurrence, distant metastasis, or death. OS was calculated as the time from surgery to death from any cause or the last follow up.

### Statistical analysis

In this study, data analysis was performed using SPSS 25.0 and R 4.0.2 statistical software. Continuous variables were expressed as mean ± standard deviation (SD) or median (interquartile range, IQR), and categorical variables were expressed as counts and percentages. The optimal NPAR cutoff was determined using Receiver Operating Characteristic (ROC) curve analysis. Kaplan–Meier survival curves and log-rank tests were used to compare survival outcomes between the low and high NPAR groups. Cox proportional hazards models were used to identify independent prognostic factors. The nomograms were constructed based on the significant variables identified in the Cox regression model using the rms package. The predictive ability of the nomograms was evaluated using the C-index and calibration curves. Internal validation was carried out by randomly dividing the cohort into a training set and a validation set in a 7:3 ratio. Decision curve analysis (DCA) was used to compare the clinical benefits of the NPAR-based nomogram with traditional TNM staging. A *p*-value < 0.05 was considered statistically significant.

## Results

### Demographic characteristics

The study included 1,439 CRC patients, with a mean age of 58.16 years. Among the cohort, 704 patients (48.9%) had colon cancer, and 735 (51.1%) had rectal cancer. Clinicopathological staging revealed that 765 (53.1%) cases were in stages I–II, while 675 (46.9%) were in stages III–IV. Perineural invasion was observed in 149 patients, and vascular invasion was present in 247 patients. The NPAR values ranged from 0.193 to 4.330, with a mean of 1.600 ± 0.397 and a median of 1.544. Pearson correlation analysis revealed a low correlation between the neutrophil percentage and albumin, with a correlation coefficient of 0.687. The optimal NPAR cutoff value was determined to be 1.62 based on ROC curve analysis, which divided the patients into a low NPAR group (< 1.62, *n* = 872) and a high NPAR group (≥1.62, *n* = 567) (Supplementary Figure S1). A high NPAR was significantly associated with advanced age, male gender, lower body mass index (BMI), advanced tumor stage, larger tumor size, and higher carcinoembryonic antigen (CEA) levels. Compared to the low NPAR group, the high NPAR group had a higher overall mortality rate (49.9% vs. 34.1%, *p* < 0.001) and recurrence rate (32.5% vs. 24.5%, *p* < 0.001) (Supplementary Table S1 and Supplementary Figure S2).

### Comparison of prognostic markers

To compare the predictive abilities of NPAR and other relevant markers for the prognosis of CRC patients, ROC curves were plotted, and the areas under the curves (AUCs) were calculated. For 3-year PFS, the AUC for NPAR was higher than that for NLR, PLR, and PNI (0.566 vs. 0.545 vs. 0.534 vs. 0.557). Similarly, for 5-year PFS, NPAR had a higher AUC compared to these markers (0.561 vs. 0.547 vs. 0.541 vs. 0.559). In terms of OS, NPAR also demonstrated better prognostic predictive efficacy than NLR, PLR, and PNI (3-year OS: 0.573 vs. 0.566 vs. 0.555 vs. 0.566; 5-year OS: 0.565 vs. 0.552 vs. 0.545 vs. 0.562; Supplementary Figure S3).

### Survival differences in low NPAR vs. high NPAR groups

**Figure 1:**
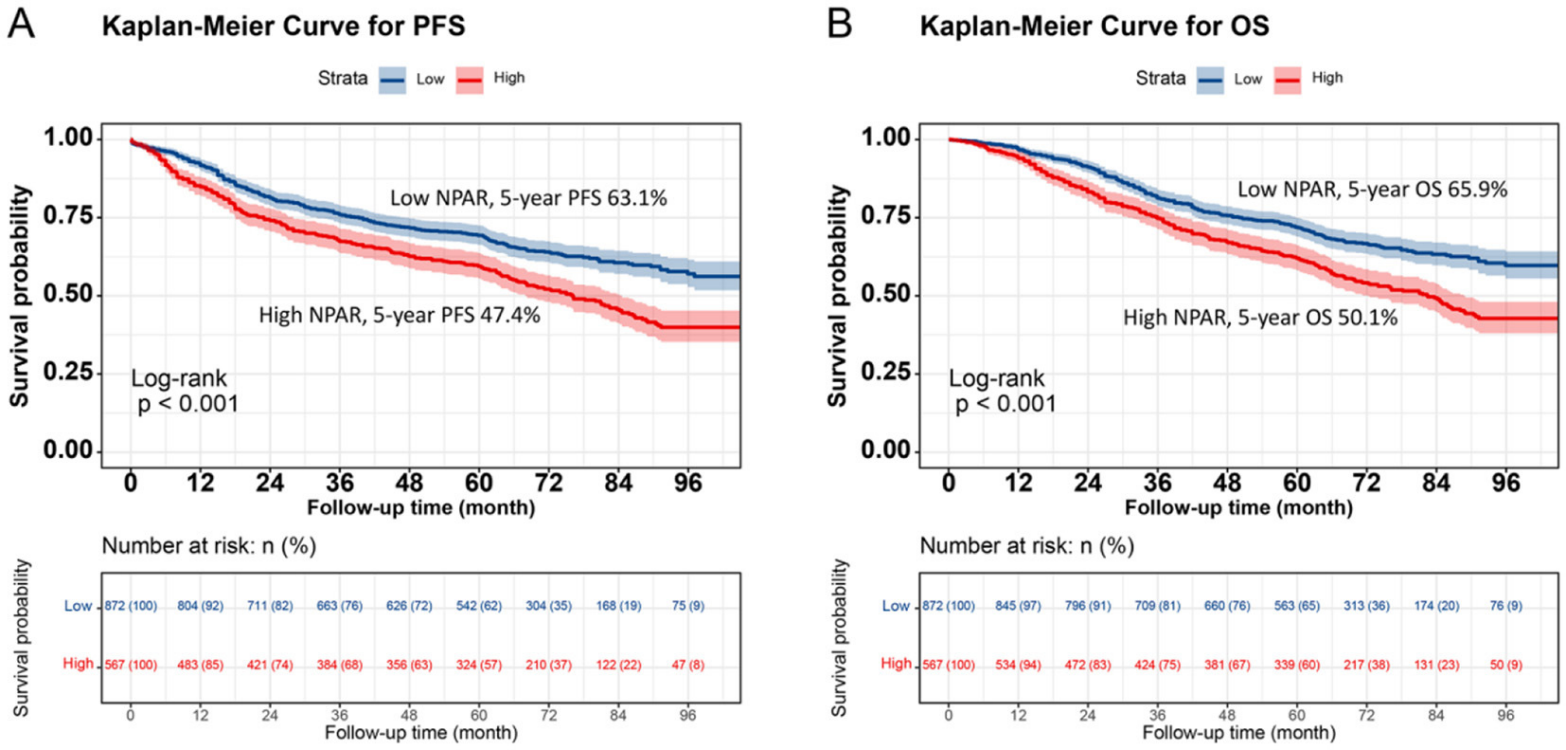
Kaplan-Meier survival analysis of PFS and OS stratified by preoperative NPAR in CRC patients. **(A)** PFS curves: high NPAR group (red, lower curve) vs. Low NPAR group (blue, upper curve). 5-year PFS rates: 47.4% (red, lower curve) vs. 63.1% (blue, upper curve) (*p* < 0.001). **(B)** OS curves: high NPAR (red, lower curve) vs. Low NPAR (blue, upper curve). 5-year OS rates: 50.1% (red, lower curve) vs. 65.9% (blue, upper curve) (*p* < 0.001). Survival probabilities were estimated using the Kaplan-Meier method, and differences were assessed by log-rank test. This figure demonstrates the prognostic value of NPAR in distinguishing survival outcomes among CRC patients.

**Figure 2:**
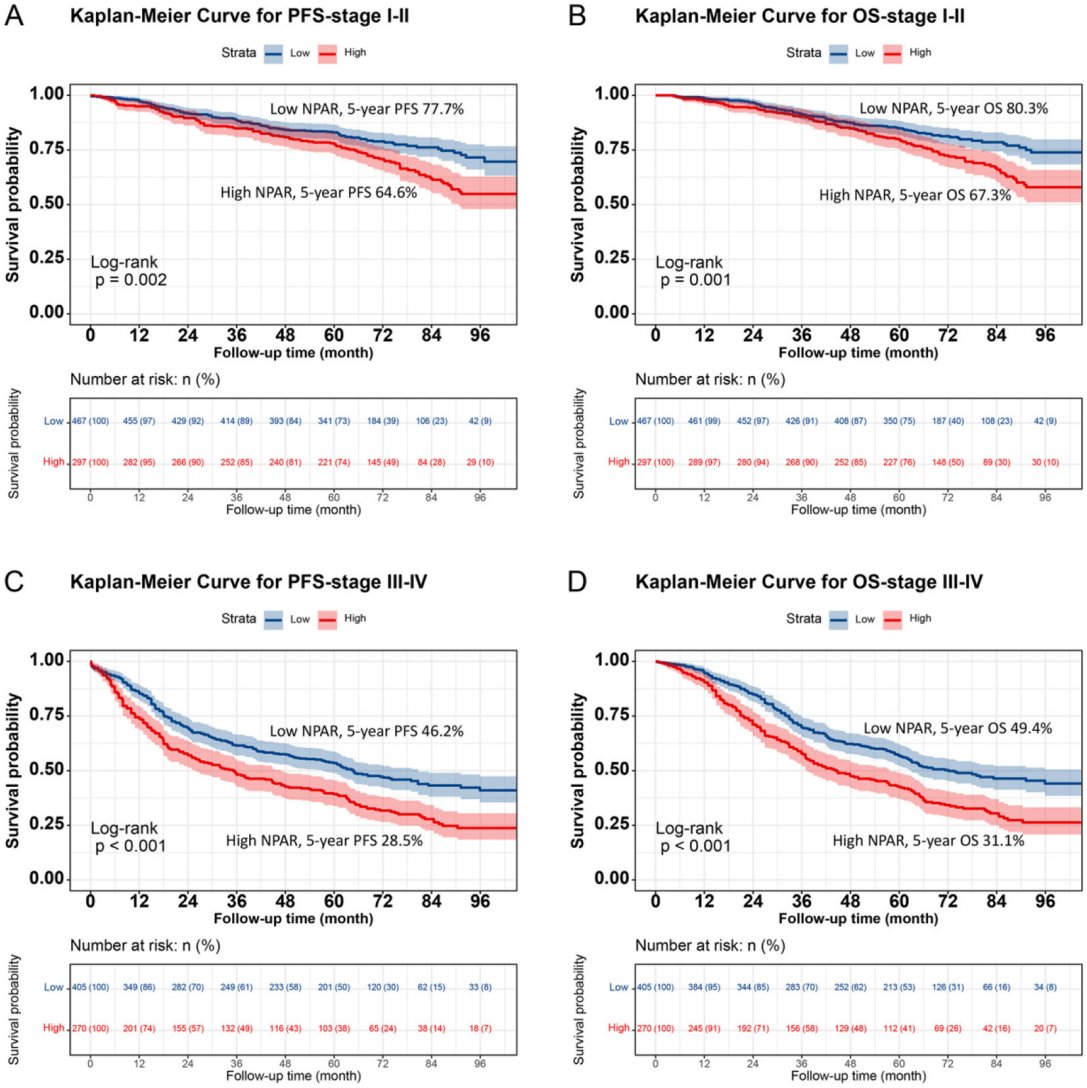
Stratified Kaplan-Meier survival curves of NPAR by TNM stage subgroups in CRC patients. **(A)** PFS for Stage I-II patients: High NPAR (red, lower curve) vs. Low NPAR (blue, upper curve). 5-year PFS rates: 64.6% (red, lower curve) vs. 77.7% (blue, upper curve) (*p* = 0.002). **(B)** OS for Stage I-II patients: High NPAR (red, lower curve) vs. Low NPAR (blue, upper curve). 5-year OS rates: 67.3% (red, lower curve) vs. 80.3% (blue, upper curve) (*p* = 0.001). **(C)** PFS for Stage III-IV patients: high NPAR (red, lower curve) vs. low NPAR (blue, upper curve). 5-year PFS rates: 28.5% (red, lower curve) vs. 46.2% (blue, upper curve) (*p* < 0.001). **(D)** OS for Stage III-IV patients: High NPAR (red, lower curve) vs. Low NPAR (blue, upper curve). 5-year OS rates: 31.1% (red, lower curve) vs. 49.4% (blue, upper curve) (*p* < 0.001). This figure highlights NPAR's prognostic utility across different tumor stages.

Kaplan–Meier analysis revealed a significant difference in prognosis between the low NPAR and high NPAR groups. Patients in the high NPAR group had a significantly lower 5-year survival rate than those in the low NPAR group (PFS: 47.4% vs. 63.1%, *p* < 0.001; OS: 50.1% vs. 65.9%, *p* < 0.001; Figures 1A, B). Subgroup analysis using Kaplan–Meier curves was also conducted. In the TNM staging subgroup, for both stages I–II and III–IV, the high NPAR group had poorer PFS than the low NPAR group (Stage I–II: 64.6% vs. 77.7%, *p* = 0.002; Stage III–IV: 28.5% vs. 46.2%, *p* < 0.001) (Figures 2A, C). Similar results were observed for OS (Stage I–II: 67.3% vs. 80.3%, *p* = 0.001; OS: 31.1% vs. 49.4%, *p* < 0.001; Figures 2B, D). For the tumor location subgroup, in colon cancer, the high NPAR group had shorter PFS and OS than the low NPAR group (PFS: 50.4% vs. 65.7%, *p* < 0.001; OS: 53.1% vs. 67.0%, *p* < 0.001; Supplementary Figure S4). In rectal cancer, patients with a high NPAR had significantly worse PFS and OS compared to those with a low NPAR (PFS: 43.0% vs. 61.2%, *p* < 0.001; OS: 45.7% vs. 65.1%, *p* < 0.001; Supplementary Figure S5). NPAR effectively differentiated between PFS and OS in both the normal and high CEA subgroups, with more significant differences in the high CEA subgroup (Supplementary Figure S6).

### Association between NPAR and survival outcomes

**Figure 3:**
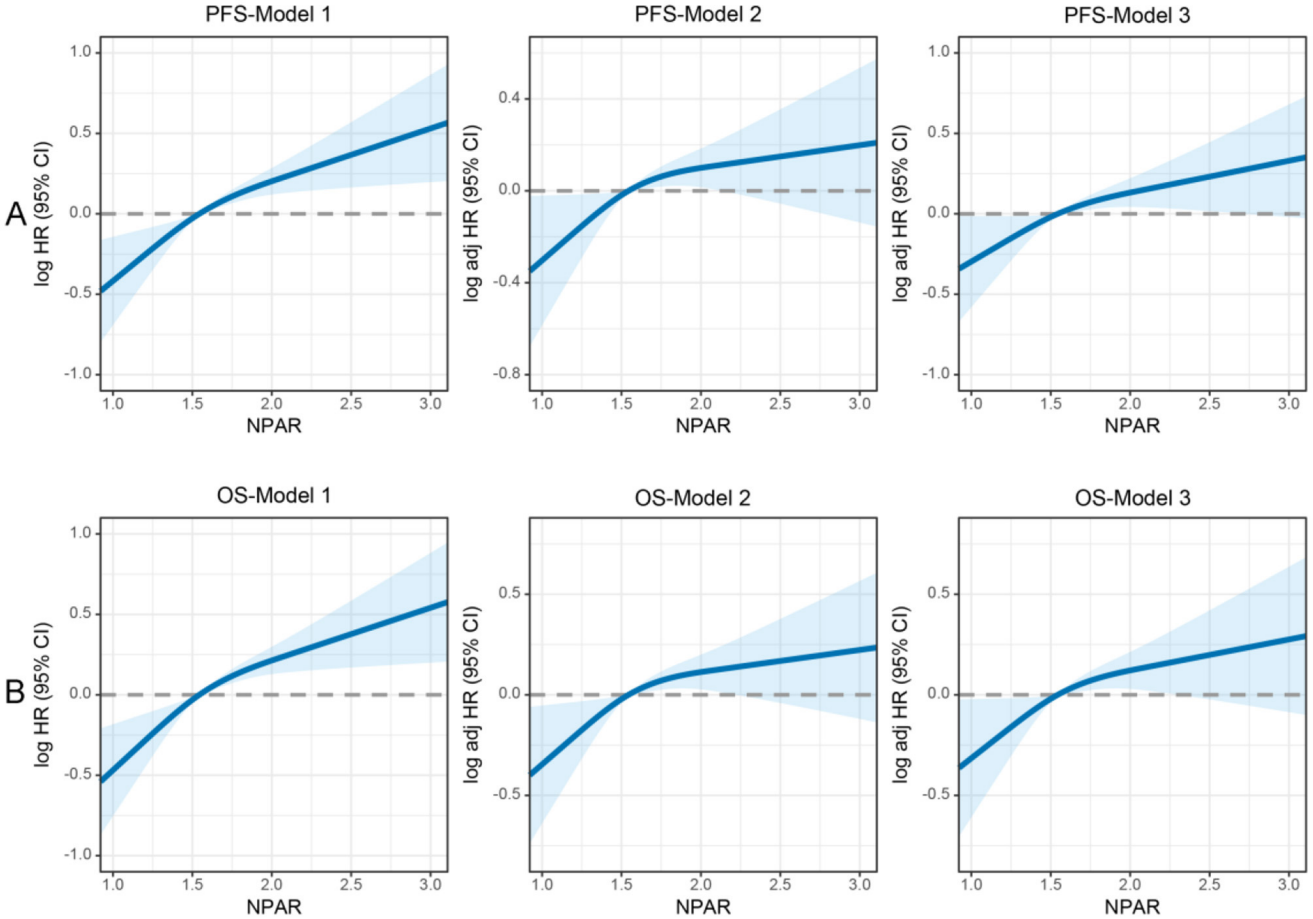
Restricted cubic spline analysis of the nonlinear association between preoperative NPAR and survival outcomes in CRC patients. **(A)** PFS and **(B)** OS. Model 1: Unadjusted. Model 2: Adjusted for gender, age, BMI, T stage, N stage, and M stage. Model 3: Fully adjusted for gender, age, BMI, hypertension, diabetes, T/N/M stage, tumor size, 0perineural/vascular invasion, differentiation, CEA, radiotherapy, and chemotherapy. RCS curves were generated using 3 knots placed at the 10th, 50th, and 90th percentiles of NPAR. Solid lines represent hazard ratios, and shaded bands indicate 95% confidence intervals. This figure highlights the nonlinear dynamics of NPAR as a continuous prognostic marker and identifies a critical threshold for clinical risk stratification in CRC patients.

A non-linear relationship was found between NPAR and PFS/OS. As the NPAR increased, the hazard ratio (HR) gradually increased. For every 1 standard deviation (SD) increase in NPAR, the risk for PFS in patients with CRC increased by 11.6% [HR = 1.116, 95% confidence interval (CI): 1.036–1.202, *p* = 0.004] (Figure 3A). Similarly, when exploring the relationship between NPAR and OS, every 1 SD increase in NPAR led to a 10.4% increase in the risk of adverse OS (HR = 1.104, 95% CI: 1.023–1.191, *p* = 0.011; Figure 3B). The high-risk group (NPAR ≥ 1.62) had a 44.0% higher risk for adverse PFS than the low-risk group (HR = 1.440, 95% CI: 1.220–1.700, *p* = 0.004). A quartile analysis of NPAR showed that patients in the second, third, and fourth quartiles had adverse PFS rates that were 0.943, 1.061, and 1.439 times higher, respectively, than those in the first quartile (Table 1). The high-risk group had a 42.0% higher risk for adverse OS than the low-risk group (HR = 1.420, 95% CI: 1.196–1.687, *p* = 0.011). As the NPAR increased, the HR for OS also gradually increased. The Q2 (0.934), Q3 (1.023), and Q4 (1.392) were associated with an increased risk of adverse OS for patients (Table 2). Multivariable forest plot analysis indicated that NPAR was an independent risk factor for the majority of patient subgroups for PFS (Supplementary Figure S7A). Similarly, for OS, patients with a high NPAR had a relatively worse prognosis than those with a low NPAR in most subgroups (Supplementary Figure S7B).

**Table 1:** Association between NPAR and PFS of CRC patients.

**NPAR**	**Model 1**	***p*-value**	**Model 2**	***p*-value**	**Model 3**	***p*-value**
Continuous (per SD)	1.202 (1.123, 1.287)	< 0.001	1.093 (1.019,1.172)	0.013	1.116 (1.036, 1.202)	0.004
Cutoff value (High)	1.515 (1.294, 1.773)	< 0.001	1.382(1.178,1.621)	< 0.001	1.44 (1.22, 1.7)	< 0.001
**Quartiles**						
Q1 (~1.34)	Ref		ref		ref	
Q2 (1.34~1.54)	1.109 (0.872, 1.41)	0.4	0.96 (0.754, 1.222)	0.739	0.943 (0.74, 1.202)	0.635
Q3 (1.54~1.77)	1.206 (0.953, 1.525)	0.12	1.064 (0.839, 1.349)	0.608	1.061 (0.835, 1.348)	0.627
Q4 (1.77~)	1.71 (1.369, 2.136)	< 0.001	1.395 (1.111, 1.751)	0.004	1.439 (1.136, 1.822)	0.003
*p* for trend		< 0.001		0.001		0.001

Model 1: no adjusted. Model 2: adjusted for gender, age, BMI, T stage, N stage, and M stage. Model 3: adjusted for gender, age, BMI, hypertension, diabetes, T stage, N stage, M stage, tumor size, perineural invasion, vascular invasion, macroscopic type, differentiation, CEA, radiotherapy, chemotherapy.

**Table 2:** Association between NPAR and OS of CRC patients.

**NPAR**	**Model a**	***p*-value**	**Model b**	***p*-value**	**Model c**	***p*-value**
Continuous (per SD)	1.213 (1.131,1.3)	< 0.001	1.102 (1.025,1.183)	0.008	1.104 (1.023,1.191)	0.011
Cutoff value (High)	1.552 (1.318,1.826)	< 0.001	1.415 (1.199,1.668)	< 0.001	1.42 (1.196,1.687)	< 0.001
**Quartiles**						
Q1 (~1.34)	ref	0.389	ref	0.705	ref	0.598
Q2 (1.34~1.54)	1.116 (0.87,1.432)	0.161	0.953 (0.741,1.224)	0.789	0.934 (0.726,1.203)	0.859
Q3 (1.54~1.77)	1.191 (0.933,1.521)	< 0.001	1.034 (0.808,1.323)	0.003	1.023 (0.797,1.312)	0.008
Q4 (1.77~)	1.76 (1.398,2.214)	< 0.001	1.422 (1.124,1.799)	0.001	1.392 (1.09,1.778)	0.003
*p* for trend		< 0.001		0.008		0.011

Model 1: no adjusted. Model 2: adjusted for gender, age, BMI, T stage, N stage, and M stage. Model 3: adjusted for gender, age, BMI, hypertension, diabetes, T stage, N stage, M stage, tumor size, perineural invasion, vascular invasion, macroscopic type, differentiation, CEA, radiotherapy, chemotherapy.

### Establishment of NPAR-based prediction nomograms

**Figure 4:**
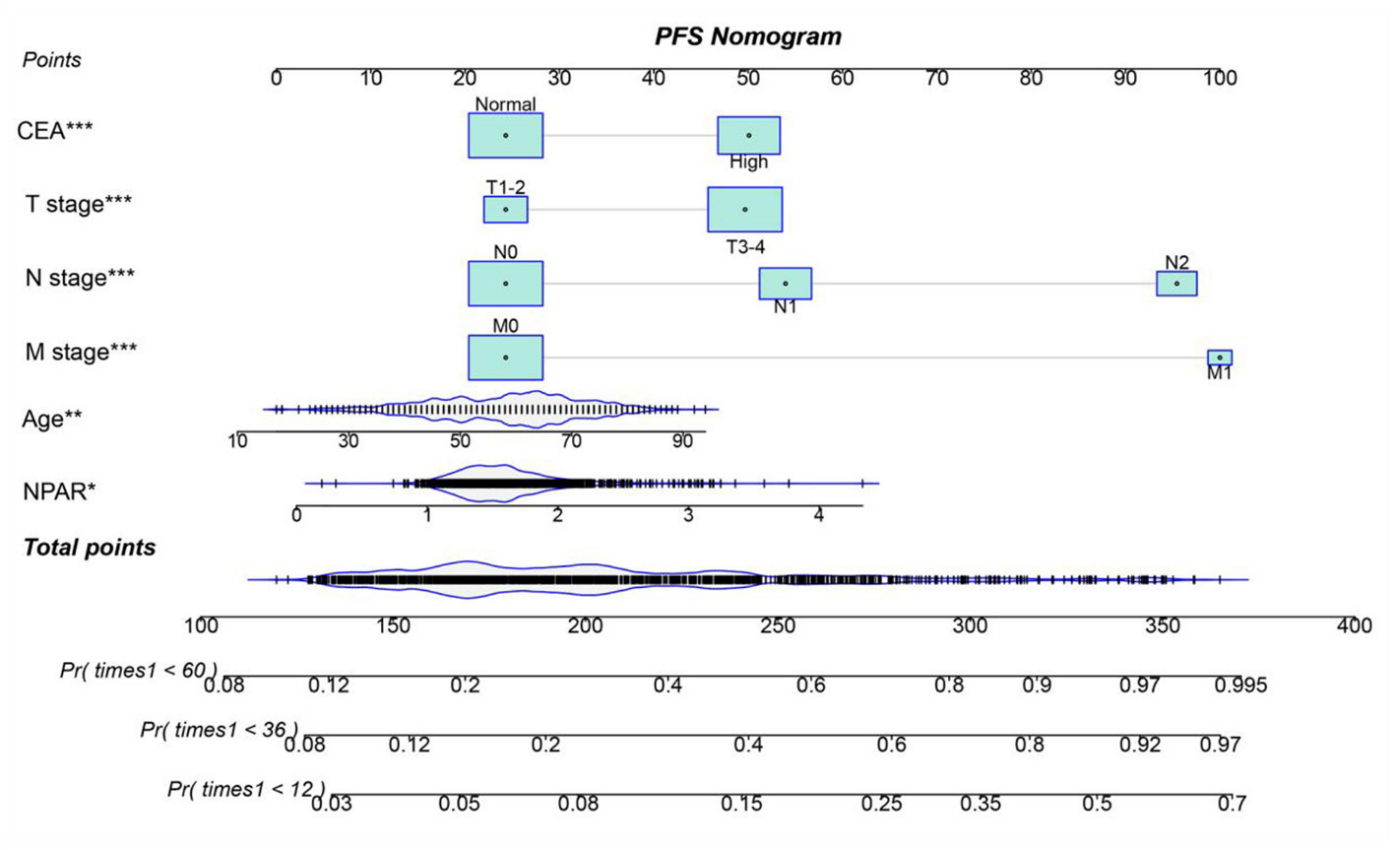
Nomogram for predicting 1-, 3-, and 5-year PFS in CRC patients. The nomogram incorporates NPAR and other independent predictors (age, T stage, N stage, M stage, CEA). Points are assigned to each variable, and total points correspond to predicted survival probabilities. The nomogram achieved a C-index of 0.721. This tool provides a visual method to estimate individualized PFS probabilities based on preoperative parameters.

**Figure 5:**
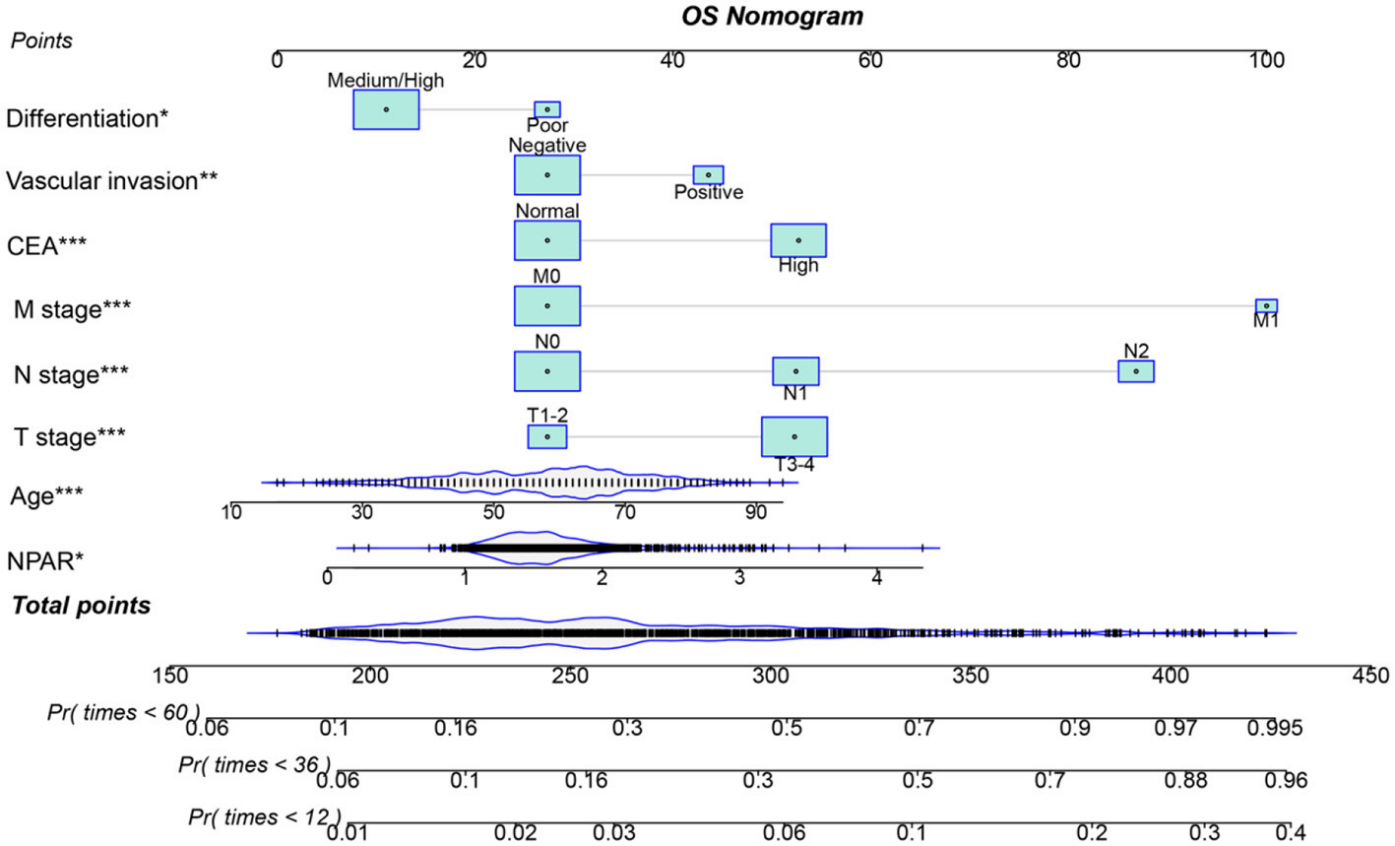
Nomogram for predicting 1-, 3-, and 5-year OS in CRC patients. The nomogram incorporates NPAR and other independent predictors (T stage, N stage, M stage, vascular invasion, differentiation, and CEA). Points are assigned to each variable, and total points correspond to predicted survival probabilities. The nomogram showed a C-index of 0.729. This figure facilitates personalized OS prediction by integrating inflammatory, nutritional, and clinicopathological factors.

Using multivariate Cox regression analysis, six independent prognostic factors that influenced PFS were identified, including age, T stage, N stage, M stage, CEA, and NPAR (Supplementary Table S2). In the multivariate Cox regression analysis of OS, T stage, N stage, M stage, vascular invasion, differentiation, CEA, and NPAR were identified as independent risk factors affecting OS in CRC patients (Supplementary Table S3). Based on these key factors, PFS/OS nomograms were constructed to predict the 1–5-year PFS and OS in CRC patients. The predicted probabilities of PFS and OS at 1, 3, and 5 years were calculated by summing the scores for each variable. Higher total scores were associated with lower PFS and OS probabilities (Figures 4, 5). The 1-, 3-, and 5-year AUCs for the PFS and OS nomograms were (1-year: 0.802, 3-year: 0.774, 5-year: 0.763) and (1-year: 0.766, 3-year: 0.775, 5-year: 0.765), respectively (Supplementary Figures S8A, B). The C-indices of the PFS and OS nomograms were 0.721 and 0.729, respectively. According to the calibration curves, there was good consistency between the actual and predicted probabilities of 1-, 3-, and 5-year PFS (Supplementary Figures S9A, B). DCA showed that the NPAR-based nomograms provided better clinical benefits than traditional tumor staging for both PFS and OS in the 1–5-year period (Supplementary Figures S10A, B). Furthermore, patients were categorized into high- and low-scoring groups based on the median scores from the nomogram. The results demonstrated that the high-score group had significantly worse PFS/OS compared to the low-score group (Supplementary Figures S11A, B).

### Validation of the NPAR-based prediction models

**Figure 6:**
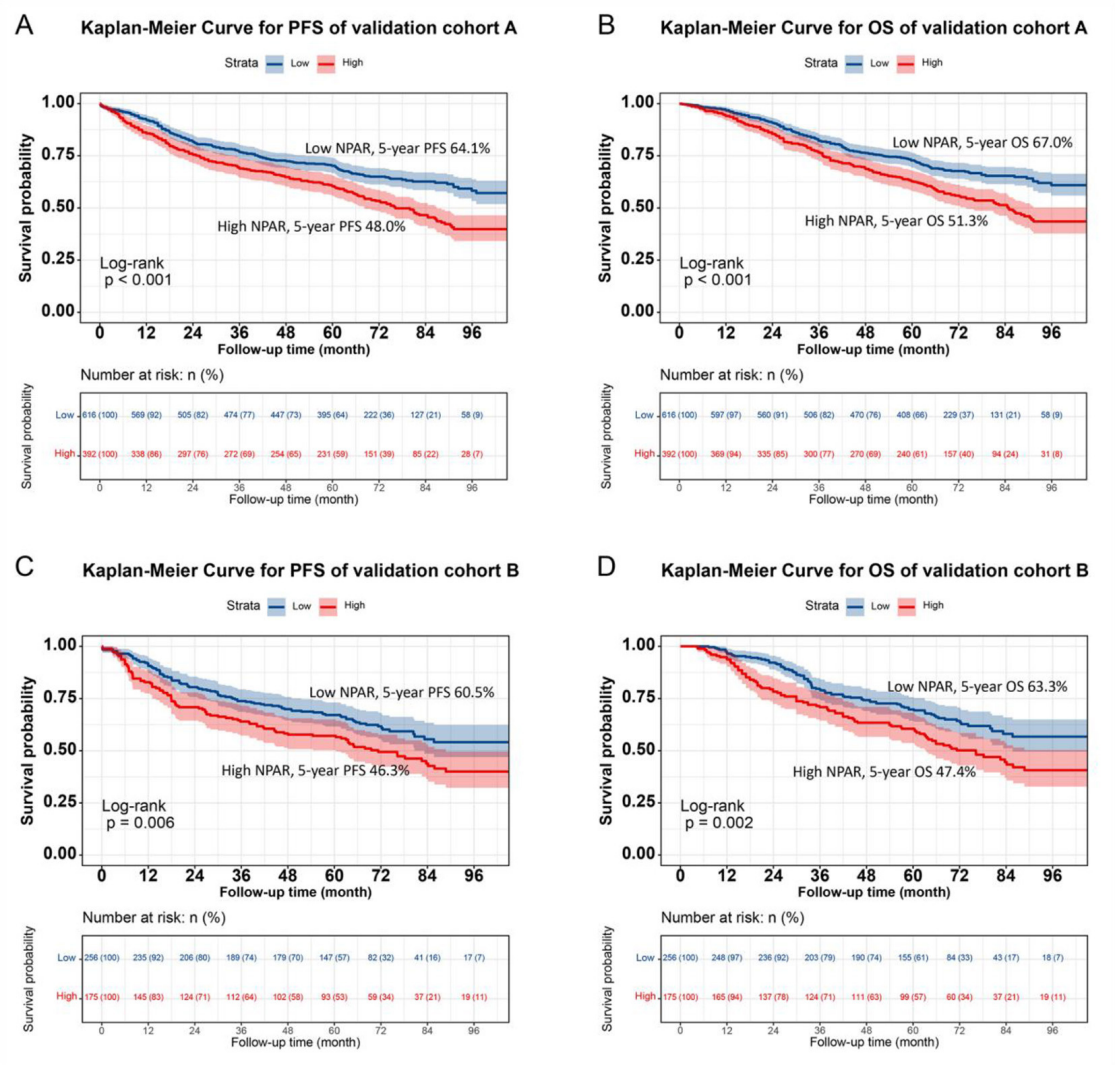
Validation of NPAR's prognostic value in validation cohort A and B using Kaplan-Meier analysis. **(A)** Progression-free survival in validation cohort A: high NPAR (red, lower curve) vs. Low NPAR (blue, upper curve). 5-year PFS rates: 48.0% (red, lower curve) vs. 64.1% (blue, upper curve) (*p* < 0.001). **(B)** Overall survival in validation cohort A: high NPAR (red, lower curve) vs. Low NPAR (blue, upper curve). 5-year OS rates: 51.3% (red, lower curve) vs. 67.0% (blue, upper curve; *p* < 0.001). **(C)** Progression-free survival in validation cohort B: high NPAR (red, lower curve) vs. Low NPAR (blue, upper curve). 5-year PFS rates: 46.3% (red, lower curve) vs. 60.5% (blue, upper curve; *p* < 0.001). **(D)** Overall survival in validation cohort B: high NPAR (red, lower curve) vs. Low NPAR (blue, upper curve). 5-year OS rates: 47.4% (red, lower curve) vs. 63.3% (blue, upper curve) (*p* < 0.001). Survival probabilities were estimated using the Kaplan-Meier method, and differences were assessed by log-rank test. This figure confirms the reproducibility of NPAR's prognostic significance in independent validation sets.

Individuals were randomly selected for internal validation in a 7:3 ratio and divided into validation cohorts A (*n* = 872) and B (*n* = 567) (Supplementary Table S4). No significant differences were observed between the two groups in terms of baseline characteristics. Overall , the results in the validation cohorts were similar to the overall results. In validation cohort A, NPAR effectively stratified the PFS/OS of CRC patients (PFS: 47.4% vs. 63.1%, *p* < 0.001; OS: 50.1% vs. 65.9%, *p* < 0.001; Figures 6A, B). In validation cohort B, patients with CRC in the high NPAR group had a lower 5-year survival rate than those in the low NPAR group (PFS: 47.4% vs. 63.1%, *p* < 0.001; OS: 50.1% vs. 65.9%, *p* < 0.001; Figures 6C, D). The C-index for PFS was 0.713 in cohort A, and 0.739 in cohort B, and for OS was 0.726 in cohort A, and 0.737 in cohort B. The 1-, 3-, and 5-year AUCs indicated that the prediction accuracy of both the PFS and OS nomograms was above 0.750 (Supplementary Figure S12). Calibration curves showed a good fit, with the predicted probabilities closely aligning with the actual observed outcomes for 1-, 3-, and 5-year survival in both validation cohorts A and B (Supplementary Figure S13). The DCA curves indicated that the NPAR-based nomogram provided greater clinical benefit compared to traditional tumor staging methods across a wide range of threshold probabilities for both PFS and OS (Supplementary Figure S14). Additionally, when patients were stratified into high- and low-scoring groups based on the nomogram scores in both validation cohorts, the high-score group consistently exhibited worse PFS and OS outcomes, further validating the predictive power of the NPAR-based nomogram (Supplementary Figure S15).

## Discussion

In the landscape of CRC research, identifying dependable prognostic indicators is crucial for customizing treatment strategies and forecasting patient outcomes. This study centered on the NPAR and explored its potential as a prognostic indicator for CRC patients, especially in relation to PFS and OS. Our findings reveal a significant correlation between NPAR and CRC prognosis. Elevated NPAR levels were strongly associated with unfavorable clinicopathological features, such as advanced age, lower BMI, advanced tumor stage, larger tumor size, and higher CEA levels. This implies that NPAR could serve as a comprehensive indicator reflecting the overall tumor host state. A high NPAR might suggest a more aggressive tumor phenotype, accompanied by an intensified inflammatory response and a compromised nutritional condition, both of which are known to fuel cancer progression.

The superiority of NPAR in prognostic prediction, compared to traditional markers like NLR, PLR, and PNI, is a remarkable discovery. NPAR's ability to integrate information on inflammation and nutrition likely accounts for its enhanced predictive power. Neutrophils play a multifaceted role in cancer. They promote tumor growth, invasion, and metastasis by releasing cytokines and proteases (23, 24, 35). Albumin, on the other hand, serves not only as a nutritional marker but also possesses anti-inflammatory and antioxidant properties (36, 37). A decline in albumin levels often indicates malnutrition and a chronic inflammatory state. Our data indicate a weak inverse correlation between the neutrophil percentage and serum albumin levels in CRC patients. This aligns with the systemic inflammatory response observed in cancer, where chronic inflammation drives neutrophilia while simultaneously suppressing albumin synthesis via cytokine-mediated pathways (e.g., IL-6 and TNF-α) (25, 36, 37). Hypoalbuminemia may further exacerbate inflammation by impairing antioxidant defenses, creating a feedback loop that sustains elevated neutrophil activity. The NPAR integrates these dynamics, reflecting both inflammatory burden (neutrophils) and nutritional status (albumin). A high NPAR signifies a pro-tumor milieu characterized by unchecked inflammation and metabolic stress, driving poor prognosis in CRC patients.

The significant disparities in PFS and OS between the high NPAR and low NPAR groups, as demonstrated by Kaplan–Meier analysis, further underscore the prognostic value of NPAR. This was consistent across various subgroups, including different TNM stages, tumor locations, and CEA levels. Notably, the more pronounced differences in advanced stage tumors highlight the potential of NPAR in guiding treatment decisions for patients with more aggressive disease. For stage III–IV CRC patients, where treatment options are often complex and challenging, NPAR can assist clinicians in stratifying patients into high- and low-risk groups. This facilitates the selection of more appropriate treatment strategies, such as intensified adjuvant therapy for high-risk patients or more conservative approaches for those with a relatively better prognosis. The identification of NPAR as an independent prognostic factor for PFS, OS, and disease recurrence through multivariable analysis solidifies its significance in CRC prognosis. For every standard deviation increase in NPAR, the heightened risk of adverse PFS and OS indicates a dose response relationship. This relationship was further corroborated by the quartile analysis, which showed a progressive increase in the risk of adverse outcomes with higher NPAR quartiles. This suggests that NPAR can be utilized as a continuous variable to stratify patients more precisely, offering a more detailed risk assessment than simple binary cut offs.

The development of the NPAR-based nomogram represents a significant advancement in personalized prognostication for CRC patients. The nomogram, which incorporates NPAR along with other established prognostic factors, demonstrated excellent predictive accuracy, as indicated by the high C-index and well-calibrated curves. This tool enables clinicians to estimate the 1-, 3-, and 5-year PFS and OS probabilities for individual patients, providing a more quantitative and intuitive approach to prognosis. In contrast to traditional tumor staging systems, which primarily rely on anatomical and pathological features, the NPAR-based nomogram takes into account the patient's inflammatory and nutritional status, offering a more comprehensive and personalized prediction. This can aid in shared decision-making between patients and clinicians, enabling more informed choices regarding treatment intensity, follow-up schedules, and supportive care.

However, this study has several limitations that need to be recognized. Firstly, the retrospective nature of the study design may introduce selection bias. The data were collected from a single institution, which may limit the generalizability of the findings. Patients in a single-center study may share similar characteristics, and the results may not be applicable to a more diverse patient population. Secondly, the relatively small sample size may have restricted the statistical power to detect subtle associations, particularly in subgroup analyses. Larger, multicenter studies are necessary to validate our findings and further explore the role of NPAR in different patient populations. Additionally, the study only measured NPAR at a single time point, preoperatively. The dynamic changes of NPAR during the course of the disease, especially in response to treatment, remain unclear. Future studies could explore the utility of serial measurements of NPAR to monitor disease progression and treatment response.

## Conclusion

This study provides evidence that NPAR is a promising prognostic indicator for CRC patients, with the potential to predict PFS and OS. The NPAR-based nomogram offers a valuable tool for individualized prognosis assessment. However, further research, particularly large-scale, multicenter, and prospective studies, is required to fully clarify the role of NPAR in CRC management and optimize its clinical application.

## Data Availability

The original contributions presented in the study are included in the article/Supplementary material, further inquiries can be directed to the corresponding authors.
