# Supplementary material for: The association between neutrophil percentage to albumin ratio and progression-free survival and overall survival in colorectal cancer patients: a retrospective cohort study
Source: Front Nutr. 2025 Jul 10;12:1589854. doi: 10.3389/fnut.2025.1589854 (PMC12286818; doi:10.3389/fnut.2025.1589854)
Supplement: Supplementary file 1 [file Data_Sheet_1.pdf]

Figure S1. Receiver operating characteristic curve for determining the optimal cutoff value of NPAR in CRC patients.

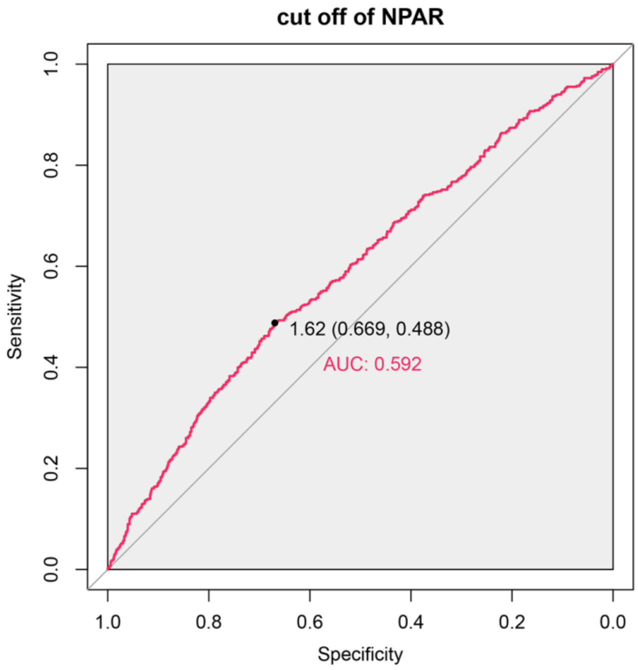

The ROC curve was generated to identify the optimal NPAR threshold for predicting 5-year overall survival (OS) using the Youden index. The area under the curve (AUC) was 0.592. A cutoff value of NPAR = 1.62 (sensitivity: 66.9%, specificity: 48.8%) was selected as the optimal threshold. This figure validates the discriminatory power of NPAR and establishes the critical value for stratifying high- and low-risk CRC patients.

Figure S2. Boxplots comparing median NPAR values across clinicopathological subgroups in CRC patients.

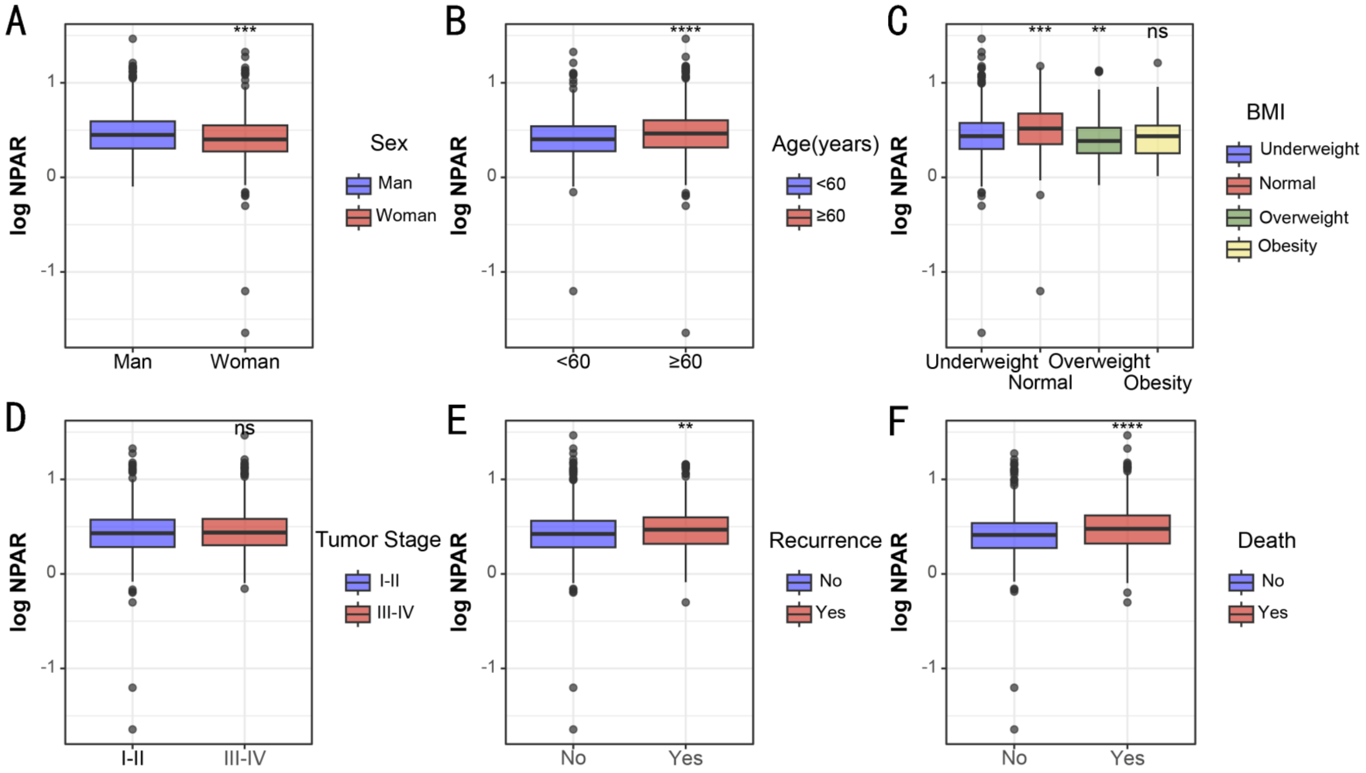

(A) Sex, (B) Age (cutoff: 60 years), (C) BMI, (D) Tumor stage (I-II vs. III-IV), (E) Recurrence status, (F) Mortality status. NPAR was significantly higher in patients with advanced tumor stage, recurrence, and mortality. This figure demonstrates the association between elevated NPAR and adverse clinicopathological features.

Figure S3. Comparison of prognostic performance between NPAR and other composite immune inflammatory

markers using ROC curves.

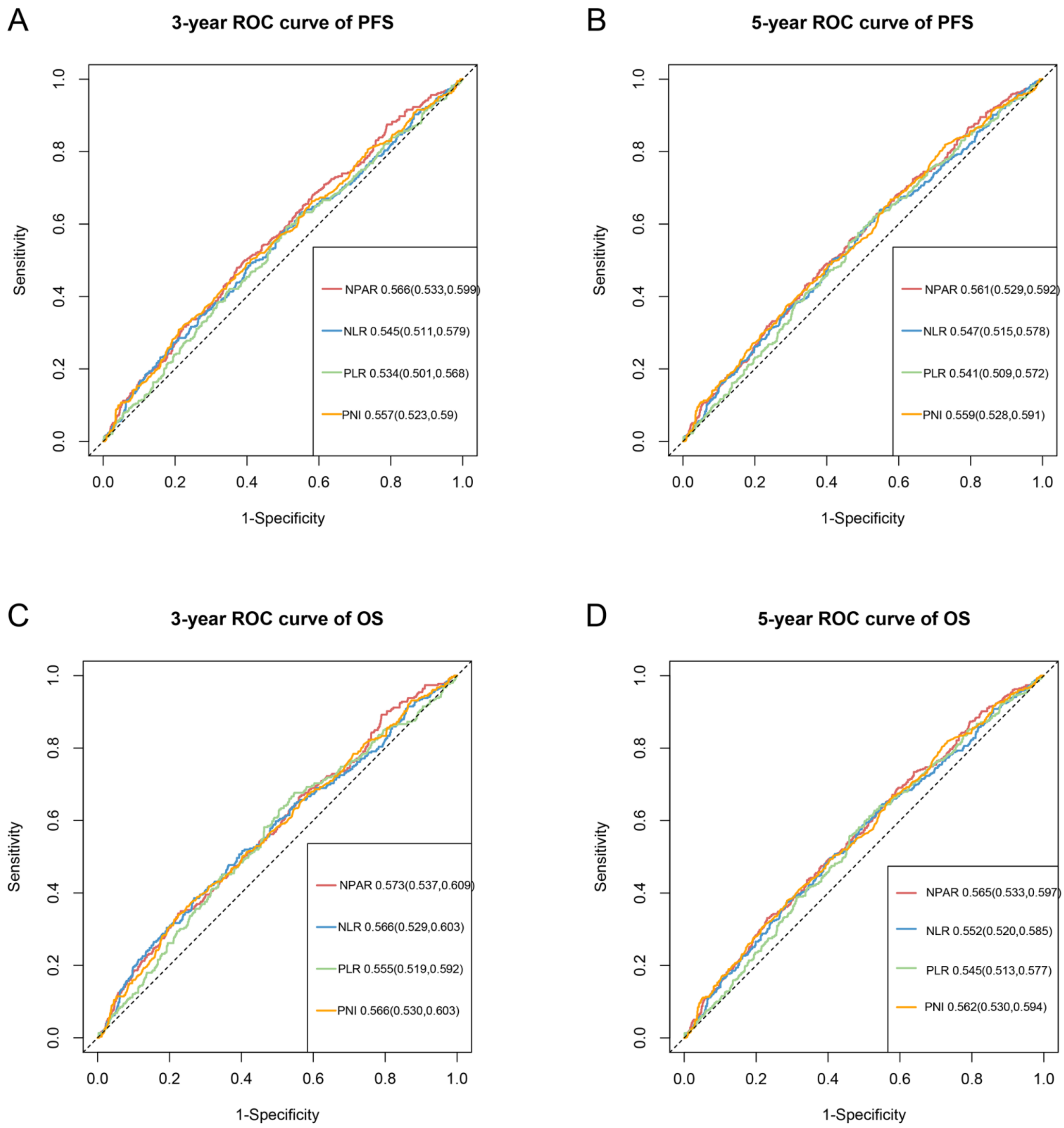

(A) 3-year PFS, (B) 5-year PFS, (C) 3-year OS, (D) 5-year OS. NPAR consistently exhibited the highest AUC values across all timepoints. This figure highlights the superior predictive accuracy of NPAR over traditional markers.

Figure S4. Kaplan-Meier survival analysis stratified by NPAR in colon cancer patients.

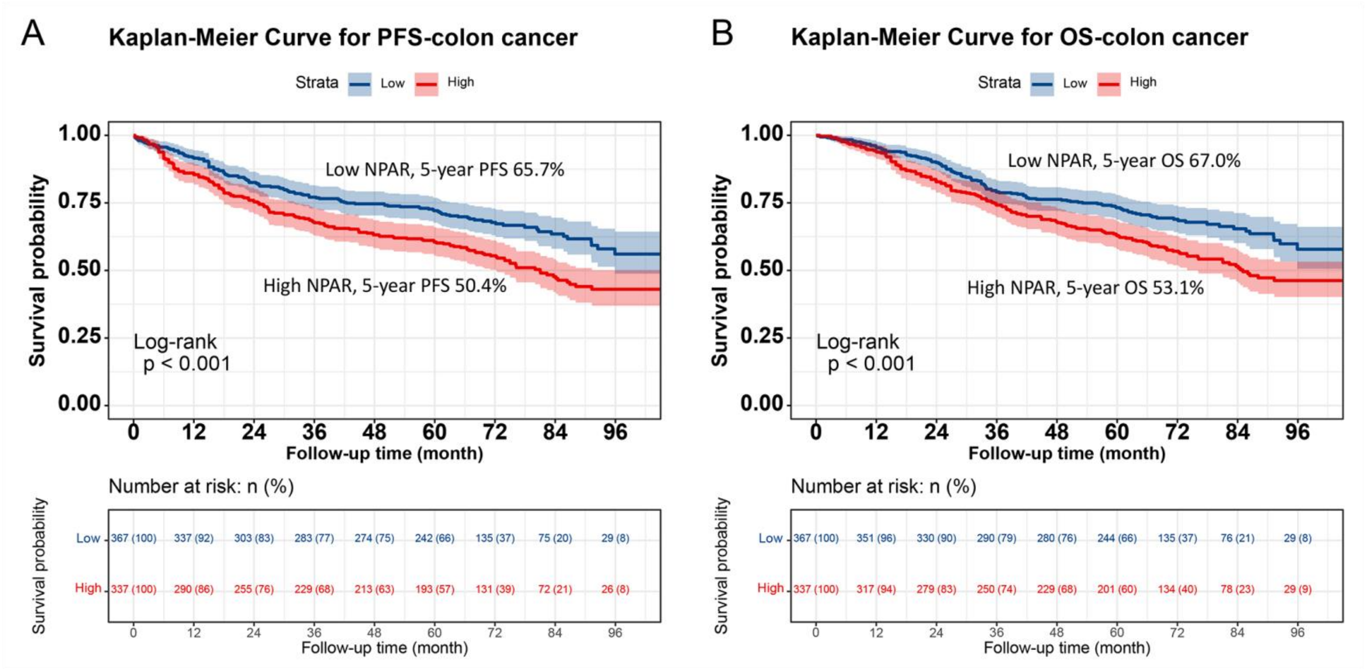

(A) PFS curves: High NPAR group (red, lower curve) vs. Low NPAR group (blue, upper curve). 5-year PFS rates: 50.4% (red, lower curve) vs. 65.7% (blue, upper curve) ($p < 0.001$).

(B) OS curves: High NPAR (red, lower curve) vs. Low NPAR (blue, upper curve). 5-year OS rates: 53.1% (red, lower curve) vs. 67.0% (blue, upper curve) ($p < 0.001$).

Survival probabilities were estimated using the Kaplan-Meier method, and differences were assessed by log-rank test. This figure confirms the prognostic value of NPAR specifically in colon cancer, independent of tumor location.

Figure S5. Kaplan-Meier survival analysis stratified by NPAR in rectal cancer patients.

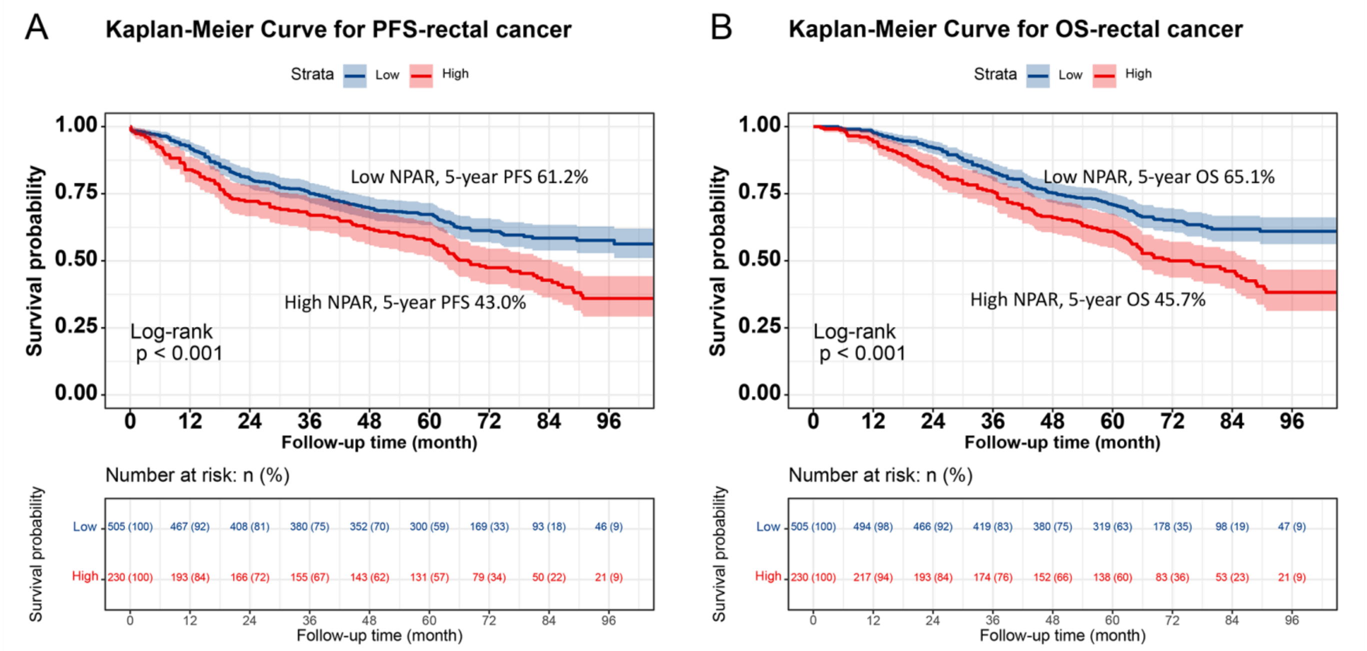

(A) PFS curves: High NPAR group (red, lower curve) vs. Low NPAR group (blue, upper curve). 5-year PFS rates: 43.0% (red, lower curve) vs. 61.2% (blue, upper curve) ($p < 0.001$).

(B) OS curves: High NPAR (red, lower curve) vs. Low NPAR (blue, upper curve). 5-year OS rates: 45.7% (red, lower curve) vs. 65.1% (blue, upper curve) ($p < 0.001$).

Survival probabilities were estimated using the Kaplan-Meier method, and differences were assessed by log-rank test. This figure confirms the prognostic value of NPAR specifically in rectal cancer, independent of tumor location.

Figure S6. Stratified Kaplan-Meier curve of NPAR based on CEA subgroup in CRC patients.

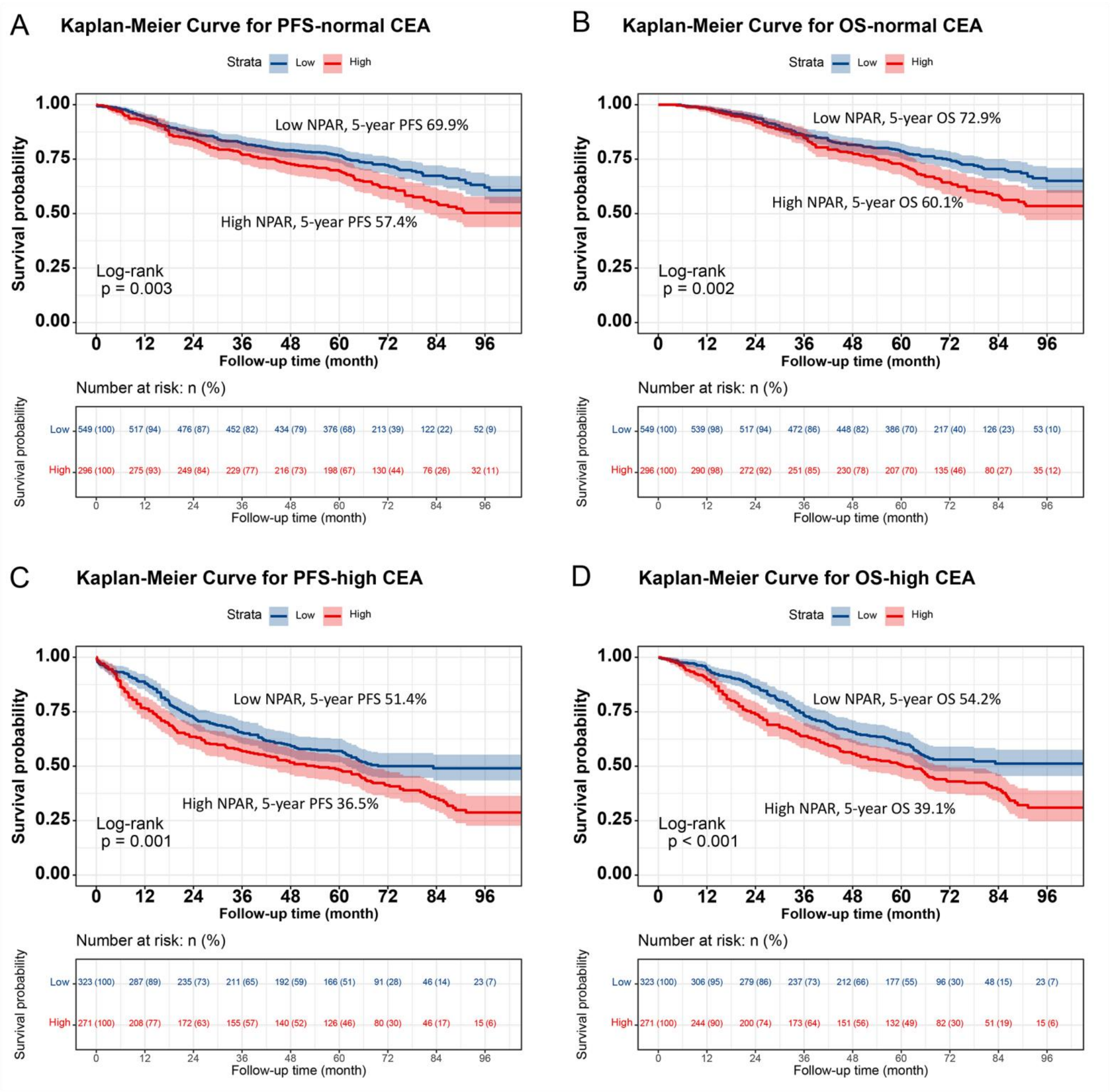

(A) PFS in the normal CEA subgroup (CEA <5 ng/mL): High NPAR group (red, lower curve) vs. Low NPAR group (blue, upper curve). 5-year PFS rates: 57.4% (red, lower curve) vs. 69.9% (blue, upper curve) ($p = 0.003$).
(B) OS in the normal CEA subgroup (CEA <5 ng/mL): High NPAR (red, lower curve) vs. Low NPAR (blue, upper curve). 5-year OS rates: 60.1% (red, lower curve) vs. 72.9% (blue, upper curve) ($p = 0.002$).
(C) PFS in the high CEA subgroup (CEA ≥5 ng/mL): High NPAR group (red, lower curve) vs. Low NPAR group (blue, upper curve). 5-year PFS rates: 36.5% (red, lower curve) vs. 51.4% (blue, upper curve) ($p = 0.001$).
(D) OS in the high CEA subgroup (CEA ≥5 ng/mL): High NPAR (red, lower curve) vs. Low NPAR (blue, upper curve). 5-year OS rates: 39.1% (red, lower curve) vs. 54.2% (blue, upper curve) ($p < 0.001$).
Survival differences were analyzed using the Kaplan-Meier method with log-rank tests. This figure demonstrates that NPAR retains prognostic significance regardless of CEA status, with more pronounced survival disparities in patients with elevated CEA levels.

Figure S7. Forest plot of subgroup analyses for the association between high NPAR and survival outcomes.

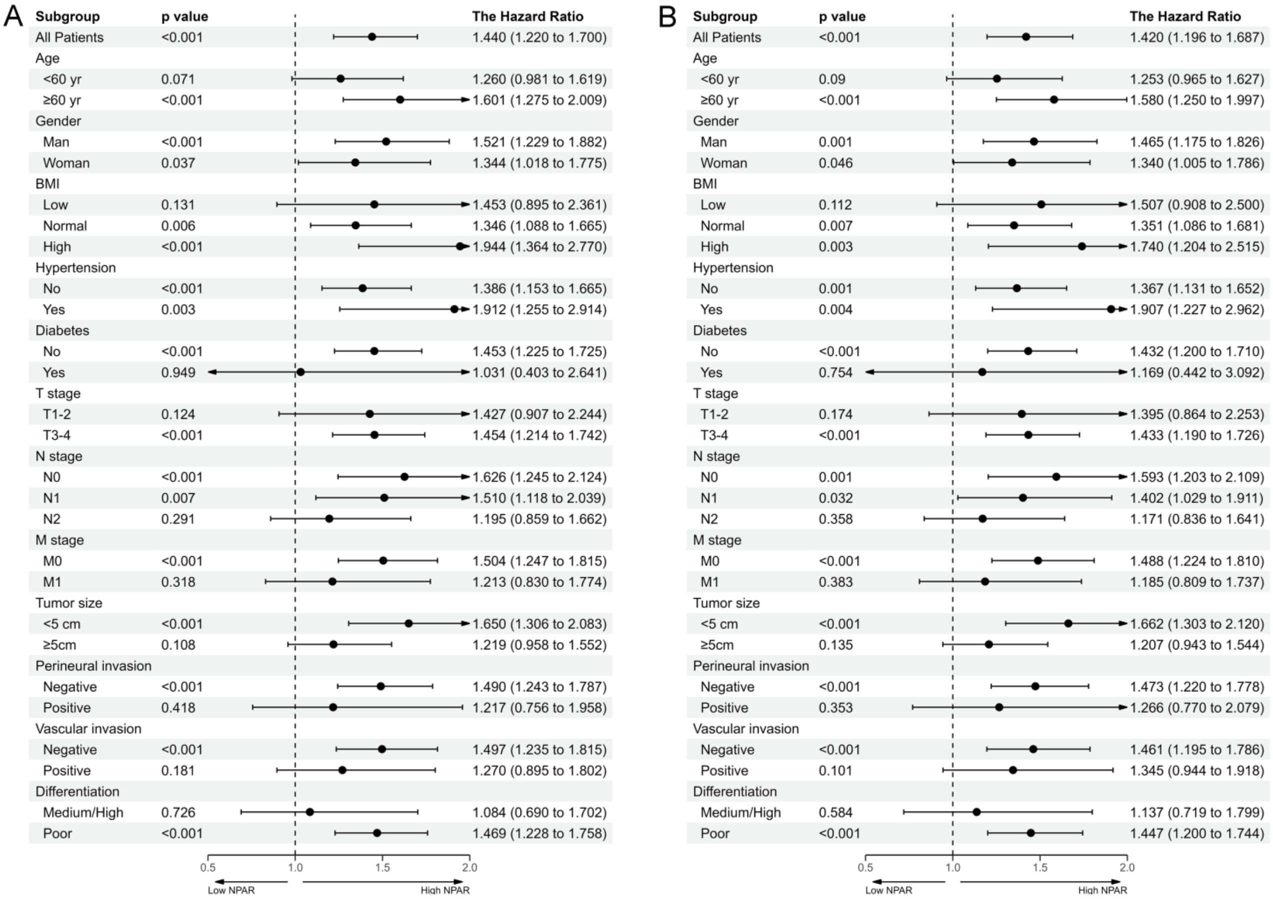

(A) PFS and (B) OS. Multivariable Cox regression adjusted for gender, age, BMI, hypertension, diabetes, T/N/M stage, tumor size, perineural/vascular invasion, differentiation, CEA, radiotherapy, and chemotherapy. High NPAR remained an independent predictor of poor survival across all subgroups. This figure validates the robustness of NPAR's prognostic significance in diverse patient subsets.

Figure S8. Time-dependent ROC curves evaluating the predictive accuracy of nomograms for PFS and OS.

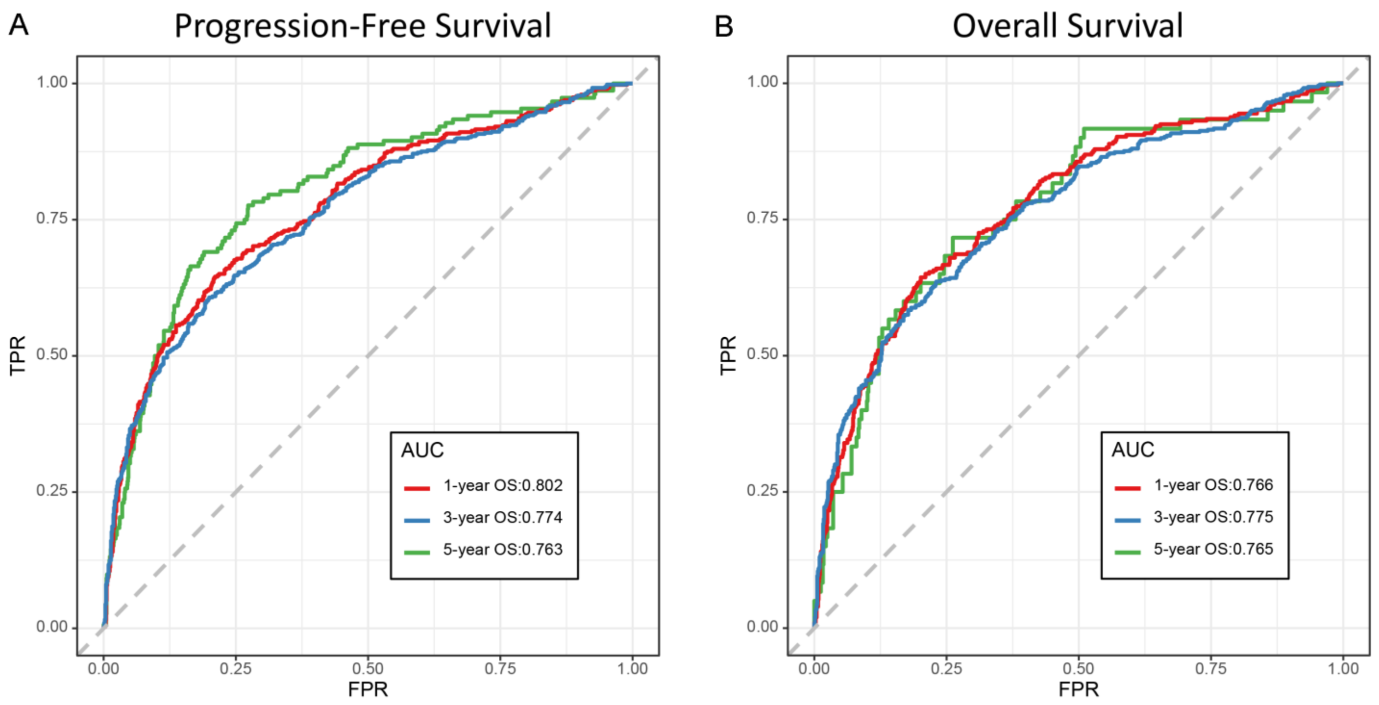

(A) PFS (1-year AUC: 0.802, 3-year: 0.774, 5-year: 0.763); (B) OS (1-year AUC: 0.766, 3-year: 0.775, 5-year: 0.765). ROC analysis was performed using the "timeROC" package in R. This figure quantifies the nomograms' discriminative ability over time, supporting their clinical utility.

Figure S9. Calibration curves assessing the accuracy of the nomograms for predicting PFS and OS in CRC patients.

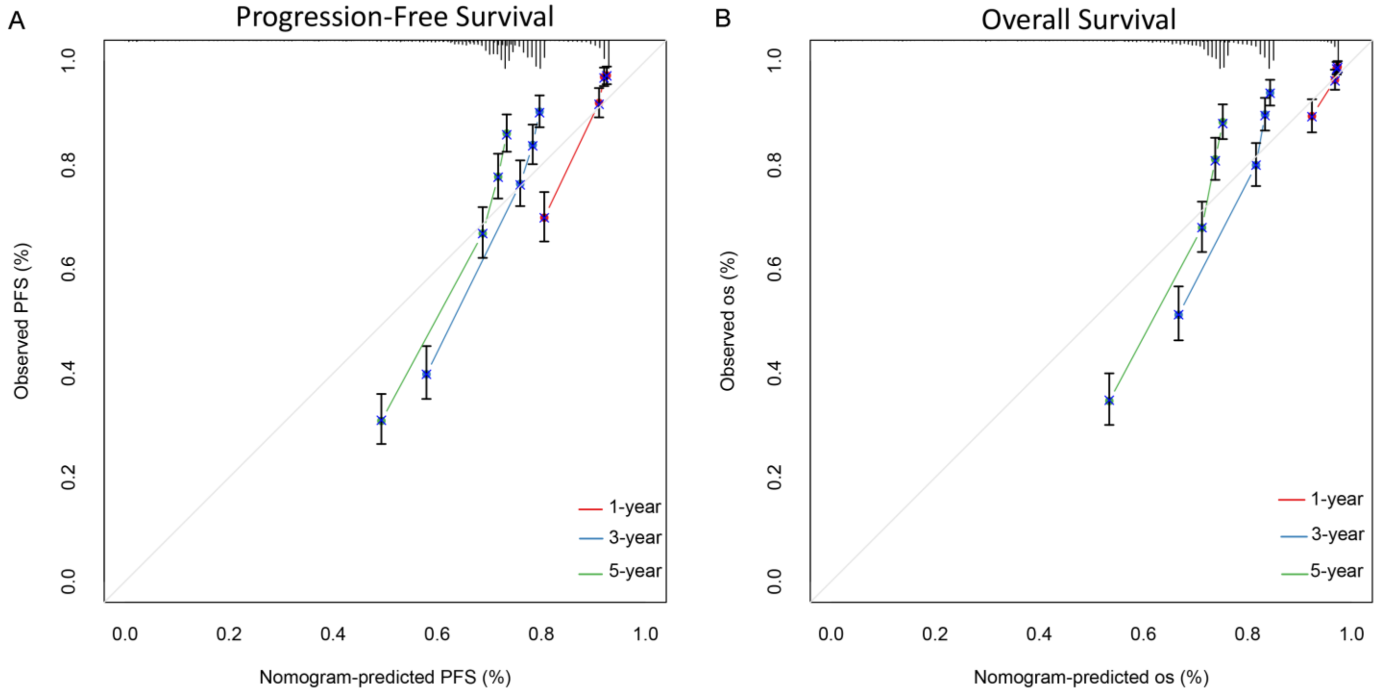

(A) PFS calibration curve, (B) OS calibration curve. Calibration was evaluated by comparing predicted survival probabilities (x-axis) with observed outcomes (y-axis) using 1,000 bootstrap resamples. The diagonal dashed line represents perfect calibration. The nomograms demonstrated good agreement between predicted and observed probabilities. This figure validates the nomograms' accuracy in predicting survival probabilities at 1-, 3-, and 5-year timepoints.

Figure S10. The DCA evaluating the clinical utility of the nomograms for predicting PFS and OS.

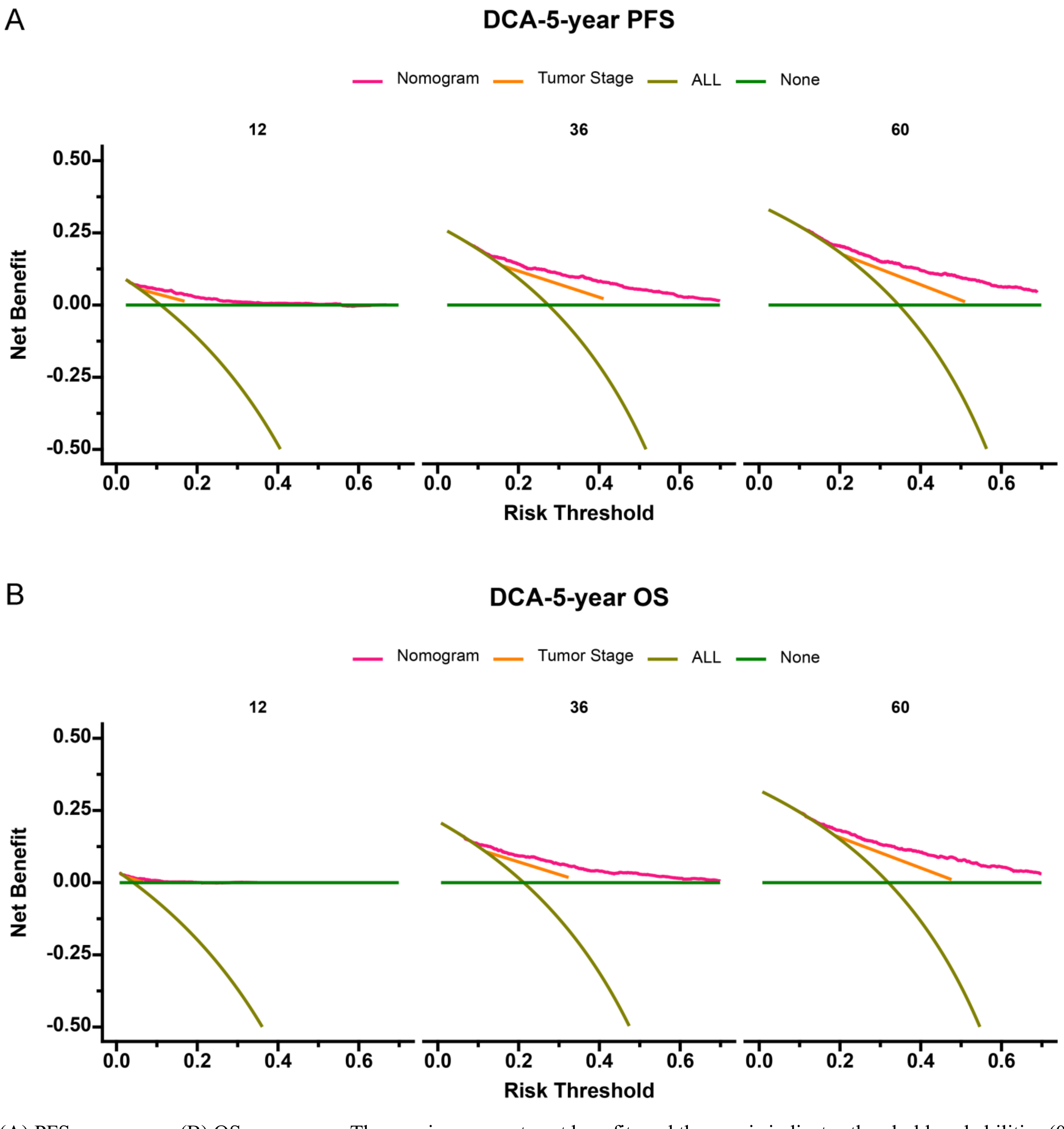

(A) PFS nomogram, (B) OS nomogram. The y-axis represents net benefit, and the x-axis indicates threshold probabilities (0-100%). The grass green line ("Treat None") and military green line ("Treat All") serve as reference strategies. The nomograms provided higher net benefit than the TNM staging system across a wide range of threshold probabilities. This figure quantifies the clinical value of the nomograms by balancing benefits and harms of interventions based on predicted risks.

Figure S11. Kaplan-Meier survival analysis stratified by nomogram risk scores in CRC patients.

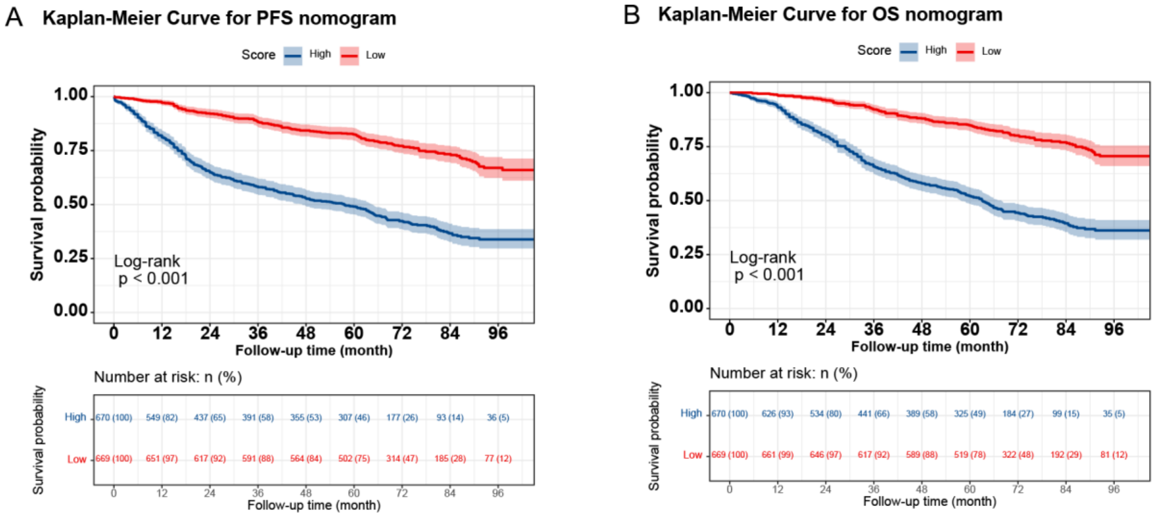

(A) PFS and (B) OS for patients categorized into high-score (blue line) and low-score (red line) groups based on the median nomogram score. High-score patients (blue, lower curve) had significantly worse 5-year PFS and OS. This figure demonstrates the nomograms' ability to risk-stratify CRC patients into distinct prognostic groups.

Figure S12. Time-dependent ROC curves for internal validation cohorts assessing nomogram performance.

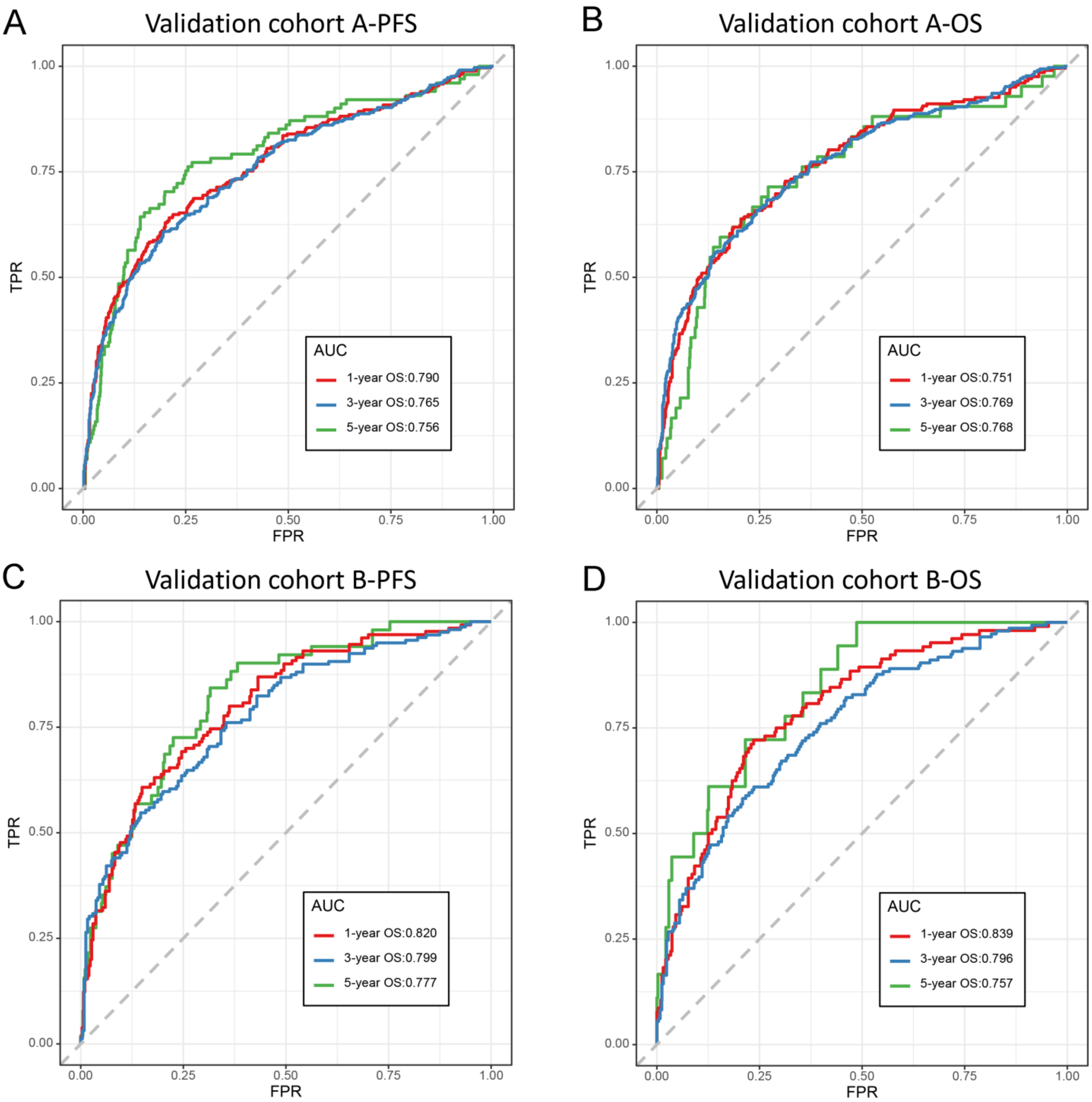

(A) PFS in cohort A, (B) OS in cohort A, (C) PFS in cohort B, (D) OS in cohort B. The nomograms maintained high predictive accuracy in both cohorts. This figure confirms the robustness of the nomograms through internal validation.

Figure S13. Calibration curves of the nomograms in internal validation cohorts.

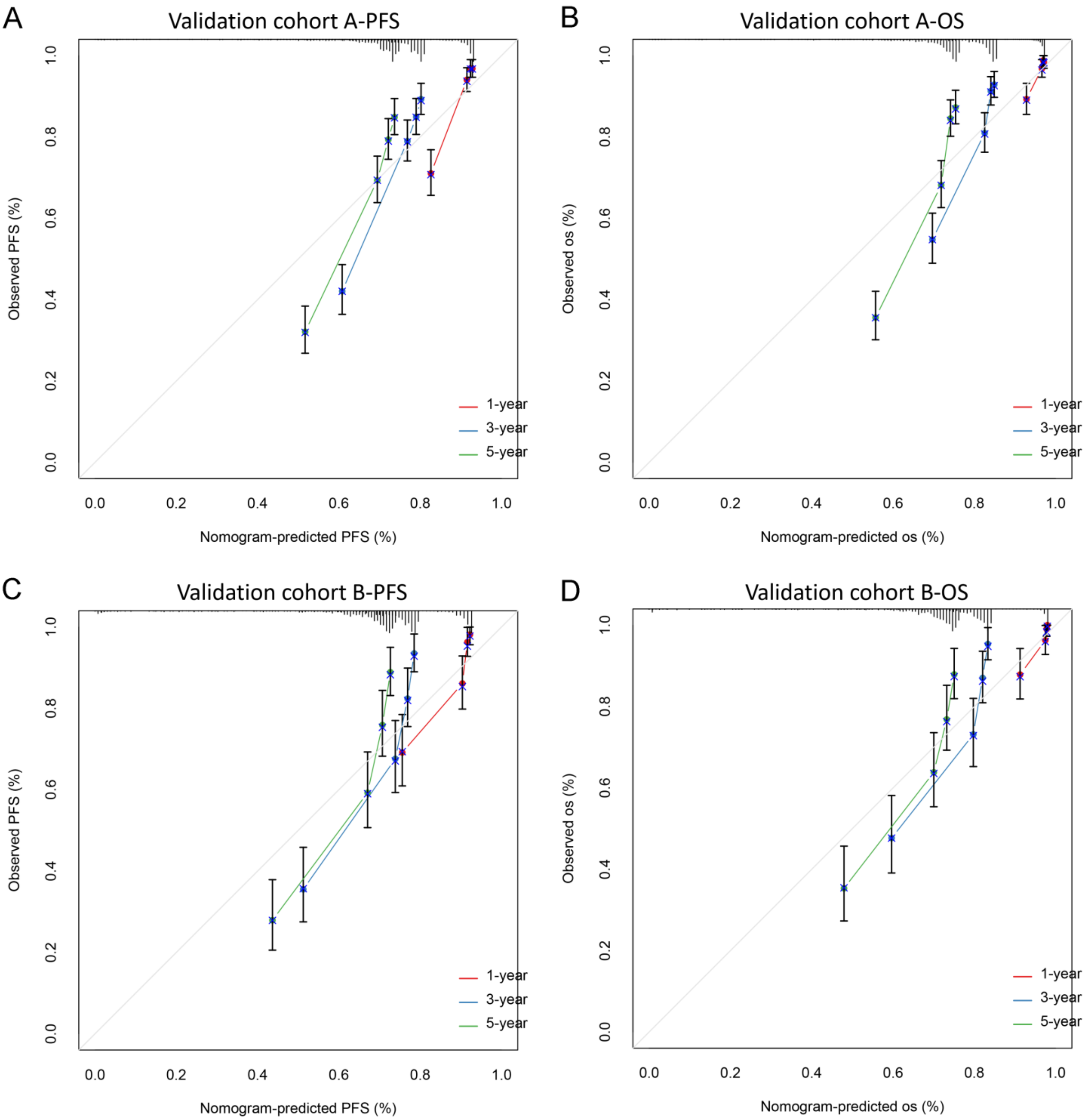

(A) PFS in cohort A, (B) OS in cohort A, (C) PFS in cohort B, (D) OS in cohort B. Calibration was assessed using the Brier score and calibration slopes. The nomograms demonstrated good agreement between predicted and observed probabilities. This figure verifies the generalizability of the nomograms' calibration across independent datasets.

Figure S14. The DCA of the nomograms in internal validation cohorts.

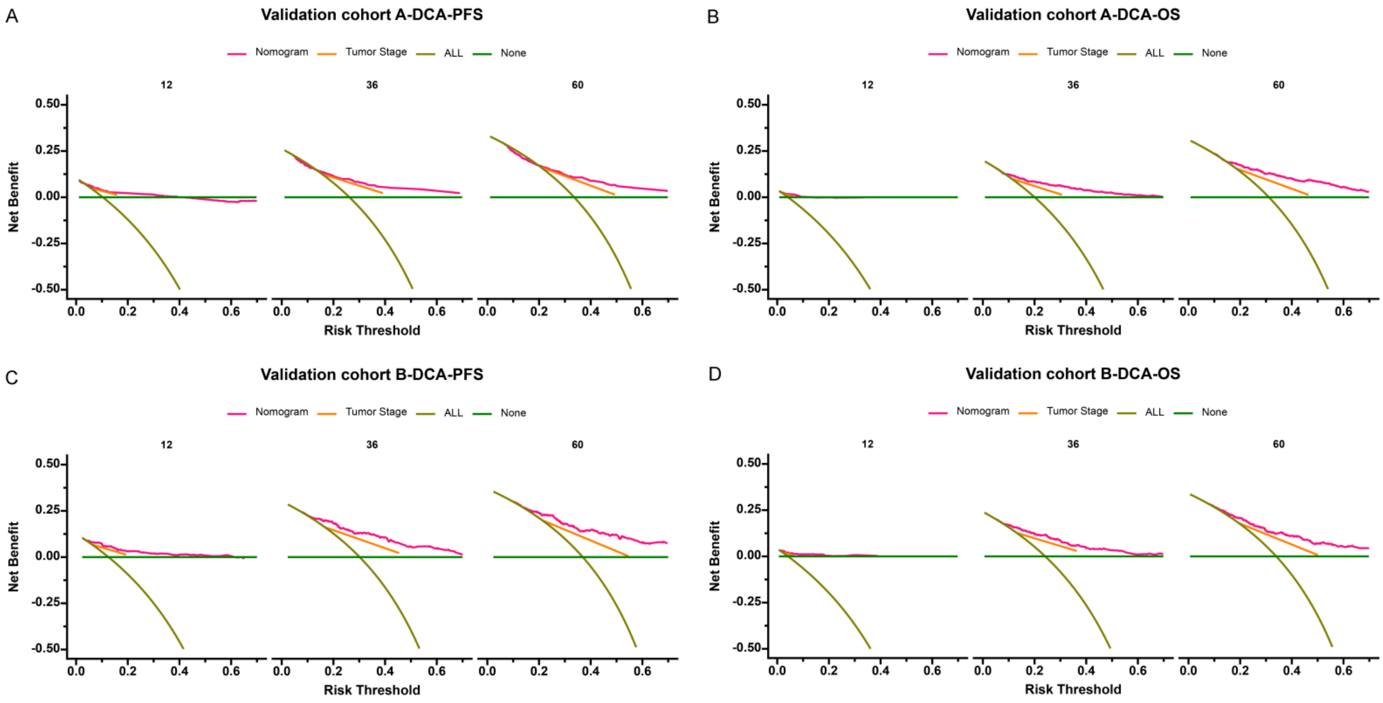

(A) PFS in cohort A, (B) OS in cohort A, (C) PFS in cohort B, (D) OS in cohort B. The nomograms consistently outperformed TNM staging in net benefit across both cohorts. This figure reinforces the clinical applicability of the nomograms in diverse patient populations.

Figure S15. Kaplan-Meier survival analysis of nomogram risk scores in internal validation cohorts.

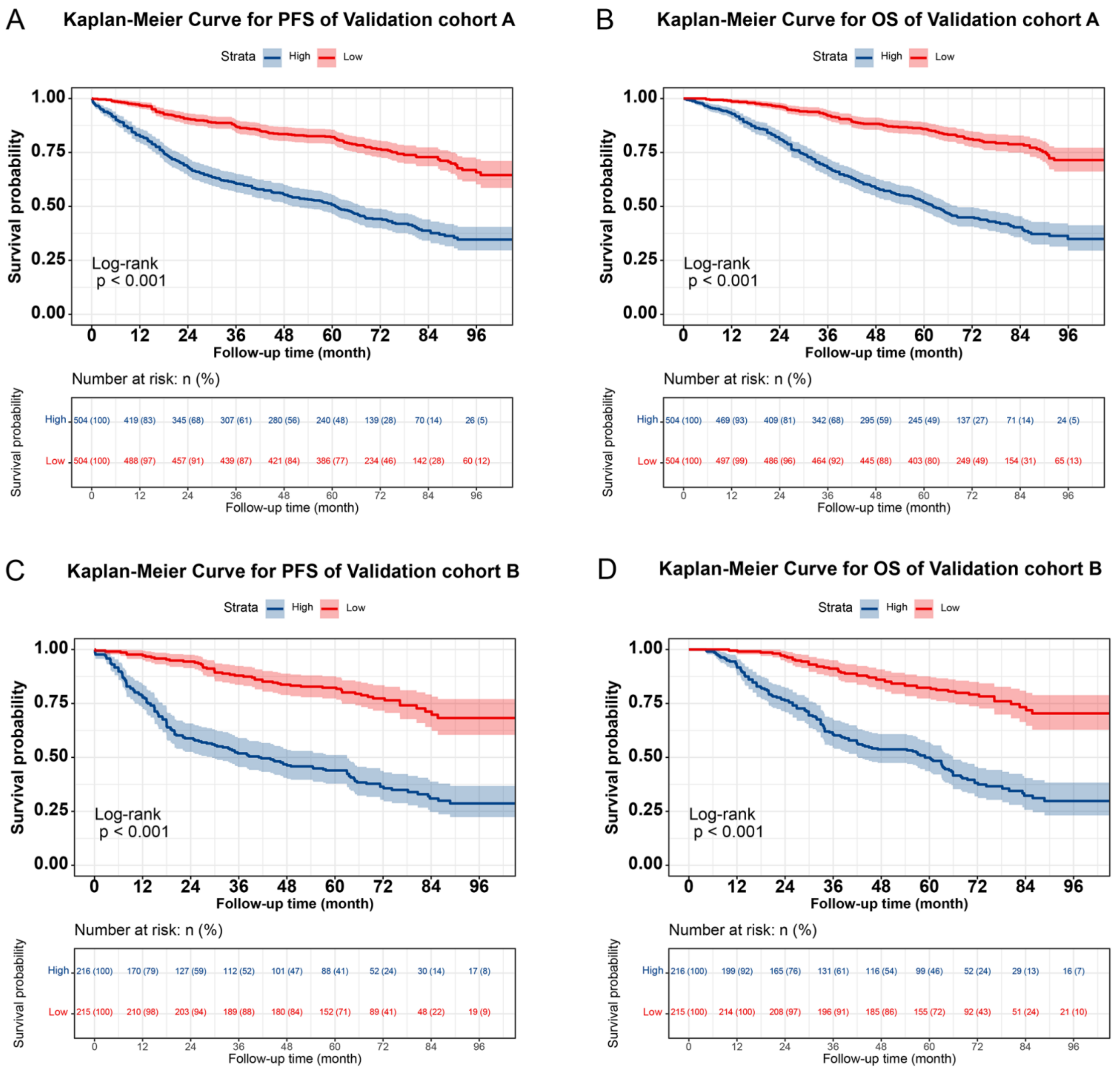

(A) PFS in cohort A, (B) OS in cohort A, (C) PFS in cohort B, (D) OS in cohort B. High-score groups (blue, lower curve) exhibited significantly poorer survival in both cohorts. This figure validates the nomograms' prognostic stratification capability in independent cohorts.

Table S1 Clinicopathological characteristics of CRC patients.

Clinicopathological characteristics	Overall	Low NPAR	High NPAR	p value
	n=1439	n=872	n=567	
Sex (Man)	903 (62.8)	518 (59.4)	385 (67.9)	0.001
Age (mean (SD))	58.16 (13.12)	56.86 (12.59)	60.16 (13.67)	<0.001
BMI (median [IQR])	22.04 [19.96, 24.33]	22.49 [20.33, 24.77]	21.38 [19.41, 23.84]	<0.001
Hypertension (Yes)	241 (16.7)	139 (15.9)	102 (18.0)	0.345
Diabetes (Yes)	90 (6.3)	48 (5.5)	42 (7.4)	0.179
T stage (T3-4)	1071 (74.4)	628 (72.0)	443 (78.1)	0.011
N stage				0.801
N0	807 (56.1)	484 (55.5)	323 (57.0)	
N1	397 (27.6)	246 (28.2)	151 (26.6)	
N2	235 (16.3)	142 (16.3)	93 (16.4)	
M stage	136 (9.5)	63 (7.2)	73 (12.9)	<0.001
TNM stage (III-IV)	675 (46.9)	405 (46.4)	270 (47.6)	0.702
Perineural invasion (Yes)	149 (10.4)	100 (11.5)	49 (8.6)	0.103
Vascular invasion (Yes)	247 (17.2)	151 (17.3)	96 (16.9)	0.906
Differentiation (Poor)	188 (13.1)	103 (11.8)	85 (15.0)	0.095
Location (Rectal cancer)	735 (51.1)	505 (57.9)	230 (40.6)	<0.001
Tumor size (median [IQR])	4.78 (2.05)	4.27 (1.64)	5.56 (2.35)	<0.001
CEA (median [IQR])	3.86 [2.06, 10.73]	3.37 [1.89, 9.24]	4.55 [2.45, 14.11]	<0.001
Radiotherapy (%)	134 (9.3)	88 (10.1)	46 (8.1)	0.242
Chemotherapy (%)	656 (45.6)	415 (47.6)	241 (42.5)	0.066
Death (Yes)	580 (40.3)	297 (34.1)	283 (49.9)	<0.001
Recurrence (Yes)	398 (27.7)	214 (24.5)	184 (32.5)	0.001
Length of stay (median [IQR])	17.00 [11.00, 21.00]	16.00 [10.00, 20.00]	18.00 [14.00, 23.00]	<0.001
Hospitalization cost (median [IQR])	49521.32 [44563.62, 55969.23]	48657.49 [44322.31, 54746.42]	51748.97 [45158.66, 59189.18]	<0.001

Table Note: CRC, colorectal cancer; BMI, body mass index.

Table S2. Univariate and multivariate Cox regression analysis of clinicopathological characteristics associated with Progression-free survival in CRC patients.

Characteristic	Progression-free survival			
	Univariate analysis		Multivariate analysis	
	HR (95%CI)	P value	HR (95%CI)	P value
Age	1.287 (1.099-1.508)	0.002	1.285 (1.092 - 1.512)	0.003
T stage	2.357 (1.891-2.939)	<0.001	1.465 (1.158 - 1.852)	0.001
N stage		<0.001		<0.001
N0	Ref.		Ref.	
N1	1.873 (1.553-2.259)	<0.001	1.556 (1.282 - 1.888)	<0.001
N2	4.07 (3.35-4.945)	<0.001	2.83 (2.283 - 3.509)	<0.001
M stage	5.368 (4.395-6.556)	<0.001	3.199 (2.587 - 3.956)	<0.001
Perineural invasion (Yes)	1.76 (1.407-2.201)	<0.001	1.13 (0.882 - 1.447)	0.333
Vascular invasion (Yes)	2.004 (1.669-2.405)	<0.001	1.232 (0.999 - 1.519)	0.055
Differentiation (high/medium)	0.71 (0.57-0.883)	0.002	0.867 (0.692 - 1.087)	0.217
Tumor size (⩾5cm)	1.187 (1.014-1.389)	0.033	0.921 (0.78 - 1.088)	0.335
CEA (≥5ng/ml)	1.999 (1.707-2.342)	<0.001	1.48 (1.25 - 1.752)	<0.001
NPAR (High)	1.515 (1.294-1.773)	<0.001	1.368 (1.16 - 1.614)	<0.001

Table Note: CRC, colorectal cancer.

Table S3 Univariate and multivariate Cox regression analysis of clinicopathological characteristics associated with overall survival in CRC patients.

Characteristic	Overall survival			
	Univariate analysis		Multivariate analysis	
	HR (95%CI)	P value	HR (95%CI)	P value
Age	1.351 (1.147-1.592)	<0.001	1.321 (1.116 - 1.564)	0.001
T stage	2.488 (1.969-3.143)	<0.001	1.508 (1.177 - 1.933)	0.001
N stage		<0.001		<0.001
N0	Ref.			
N1	1.879 (1.547-2.283)	<0.001	1.548 (1.266 - 1.893)	<0.001
N2	4.096 (3.352-5.006)	<0.001	2.712 (2.172 - 3.386)	<0.001
M stage	5.578 (4.554-6.833)	<0.001	3.322 (2.679 - 4.119)	<0.001
Perineural invasion (Yes)	1.718 (1.363-2.166)	<0.001	1.075 (0.832 - 1.39)	0.581
Vascular invasion (Yes)	2.046 (1.696-2.467)	<0.001	1.278 (1.031 - 1.584)	0.025
Differentiation (high/medium)	0.657 (0.527-0.82)	<0.001	0.786 (0.625 - 0.988)	0.039
Tumor size (⩾5cm)	1.302 (1.106-1.532)	0.001	1.028 (0.866 - 1.221)	0.749
CEA (≥5ng/ml)	2.048 (1.739-2.412)	<0.001	1.478 (1.242 - 1.76)	<0.001
NPAR (High)	1.552 (1.318-1.826)	<0.001	1.361 (1.147 - 1.613)	<0.001

Table Note: CRC, colorectal cancer.

Table S4 Clinicopathological characteristics of validation cohorts

Clinicopathological characteristics	Validation cohort A n=1008	Validation cohort B n=431	p value
Sex (Man)	642 (63.7)	261 (60.6)	0.286
Age (mean (SD))	58.39 (13.22)	57.63 (12.88)	0.318
BMI (median [IQR])	22.06 [19.96, 24.47]	21.93 [19.99, 24.22]	0.442
Hypertension (Yes)	172 (17.1)	69 (16.0)	0.679
Diabetes (Yes)	60 (6.0)	30 (7.0)	0.545
T stage (T3-4)	746 (74.0)	325 (75.4)	0.624
N stage			0.531
N0	575 (57.0)	232 (53.8)	
N1	272 (27.0)	125 (29.0)	
N2	161 (16.0)	74 (17.2)	
M stage	91 (9.0)	45 (10.4)	0.459
TNM stage (III-IV)	466 (46.2)	209 (48.5)	0.466
Perineural invasion (Yes)	106 (10.5)	43 (10.0)	0.831
Vascular invasion (Yes)	180 (17.9)	67 (15.5)	0.323
Differentiation (Poor)	134 (13.3)	54 (12.5)	0.757
Location (Rectal cancer)	514 (51.0)	221 (51.3)	0.967
Tumor size (median [IQR])	4.72 (2.05)	4.92 (2.03)	0.092
CEA (median [IQR])	3.71 [1.96, 10.80]	4.07 [2.33, 10.36]	0.246
Radiotherapy (%)	98 (9.7)	36 (8.4)	0.472
Chemotherapy (%)	461 (45.7)	195 (45.2)	0.91
Death (Yes)	394 (39.1)	186 (43.2)	0.167
Length of stay (median [IQR])	17.00 [11.00, 21.00]	17.00 [11.00, 21.00]	0.58
Hospitalization cost (median [IQR])	49437.74 [44704.35, 55911.01]	49692.00 [44056.20, 56134.56]	0.872

Table Note: CRC, colorectal cancer; BMI, body mass index.
